# Tafel slopes and exchange current densities of oxygen reduction and hydrogen evolution on steel

**DOI:** 10.1177/1478422X241227829

**Published:** 2024-01-30

**Authors:** M. C. van Ede, U. Angst

**Affiliations:** 1 Institute for Building Materials, 27219 ETH Zurich, Zurich, Switzerland

**Keywords:** Kinetic parameters, Tafel slope, exchange current density, steel corrosion, ORR, HER, potentiodynamic scan

## Abstract

The prediction and prevention of steel corrosion in engineering applications rely on the accurate understanding of kinetic parameters, such as the Tafel slopes and exchange current densities. These parameters show a large spread in literature. We investigated the dependency of these kinetic parameters on the measurement methodology for stainless and carbon steels, in a controlled rotating disk electrode setup with a near-neutral (pH 7.5) buffer solution. Consistent results were found for hydrogen evolution on stainless steel, with Tafel slopes of −0.13 to −0.15 V/dec and exchange current densities around 0.01–0.02 A/m^2^. The studied oxygen reduction kinetics showed the largest dependency on the measurement methodology, especially the potentiodynamic scan direction. Supported by active light reflectance spectroscopy, the large observed variations were attributed to the influence of an oxide film, which may overshadow the oxygen reduction at small over-potentials. The obtained variation gives insight on the accuracy of documented and measured values.

## Introduction

The process of corrosion of steel depends on the complex interaction of the material, environment and reactions taking place at the steel–electrolyte interface. Explaining degradation of steel structures, predicting and preventing future degradation and even the development of techniques to locate and quantify occurring corrosion, rely on the understanding of the thermodynamic and kinetic fundamentals. Numerous applications require accurate values for kinetic parameters, such as the Tafel slope and exchange current density.

One example is the numerical modelling of steel corrosion, for which the accurate understanding of the corrosion kinetics becomes increasingly important. The kinetic parameters are used to describe the relation between the current density and electrical potential at the steel–electrolyte interface. This is valuable in fields were studying corrosion in situ is difficult, such as tribocorrosion,^1^ and the corrosion of steel in porous media, such as reinforced concrete^2–5^ and underground steel structures.^6–8^ The use of numerical modelling to create digital twins also gained increasing popularity in the recent years. Digital twins may be used to optimise the system design in fields such as cathodic protection.^9,10^ The choice for used values of kinetic parameters in these numerical models is often poorly justified, or authors explicitly state that the values do not represent a specific physical system. Few studies do compare experimental with modelled data.^11,12^ However, earlier works show that the electrical potential field, the current distribution and the corrosion rate obtained from numerical modelling, are very sensitive to the kinetic parameters.^5,13^ Numerical simulations and digital twins can only give accurate predictions, if the chosen kinetic parameters reflect reality. Finally, Tafel slopes are also needed in the widely used approach to estimate corrosion rates from polarisation resistance measurements. Here the Tafel slopes relate the polarisation resistance to the corrosion rate in the Stern-Geary equation.^14^

Finding accurate values for kinetic parameters such as Tafel slopes and exchange current densities is difficult. For more complicated geometries and electrolytes, such as the example of localised corrosion in reinforced concrete, Tafel slopes and exchange current densities are scarce, as their measurement is challenging and often unreliable. The measured parameters can vary greatly due to variations in ohmic drop related to reference electrode placement, changing resistivity of the concrete over time, and varying chloride content.^15–19^ However, even documented kinetic parameters for steel in solution vary greatly in the literature, as highlighted by the authors in earlier work.^20^ Both the data analysis as well as the measurement methodology can influence the determined values.

The question is how much we can rely on documented parameters in literature, or even on our own measurement of the Tafel slopes and exchange current densities, to accurately reflect the studied corrosion process. In this study we investigate the expected variation of these parameters when measured in controlled conditions. We measure Tafel slopes and exchange current densities for oxygen reduction and hydrogen evolution on stainless and carbon steel rotating disk electrodes in a near-neutral (pH 7.5) buffered electrolyte. We vary the hydrodynamic condition (rotation rate), the time of exposure to the electrolyte prior to starting the potentiodynamic scan measurements, the scan direction and the scan rate. The results of this systematic study is expected to give insight on the accuracy of documented and measured values and a starting point to estimate the actual accuracy of numerical modelling in the investigation and prediction of corrosion of steel structures.

### Corrosion kinetics of steel

The corrosion of steel is governed by the oxidation of iron, and depending on the environment, by the reduction of dissolved oxygen in the electrolyte or by the hydrogen evolution reaction (HER). The HER is the reduction of a proton, forming hydrogen:^21,22^

(1) $$2H^{+}+2e^{-}\to H_{2}$$

This reaction consists of several elementary reaction steps, starting with a single electron transfer step of $H^{+}$ to $H_{ad}$ (Volmer reaction step), followed by $H_{ad}+H_{ad}\to H_{2}$ (Tafel reaction step) or $H_{ad}+H^{+}+e^{-}\to H_{2}$ (Heyrovsky reaction step).^23^ In neutral solutions, the hydrogen is produced by water decomposition and therefore the HER becomes:^22^

(2) $$2H_{2}O+2e^{-}\to H_{2}+2OH^{-}$$

If dissolved oxygen is present in the electrolyte, the main reduction reaction is that of oxygen. In a neutral environment the oxygen reduction reaction (ORR) is given by:^21^

(3) $$O_{2}+2H_{2}O+4e^{-}\to 4OH^{-}$$

**Figure 1.**
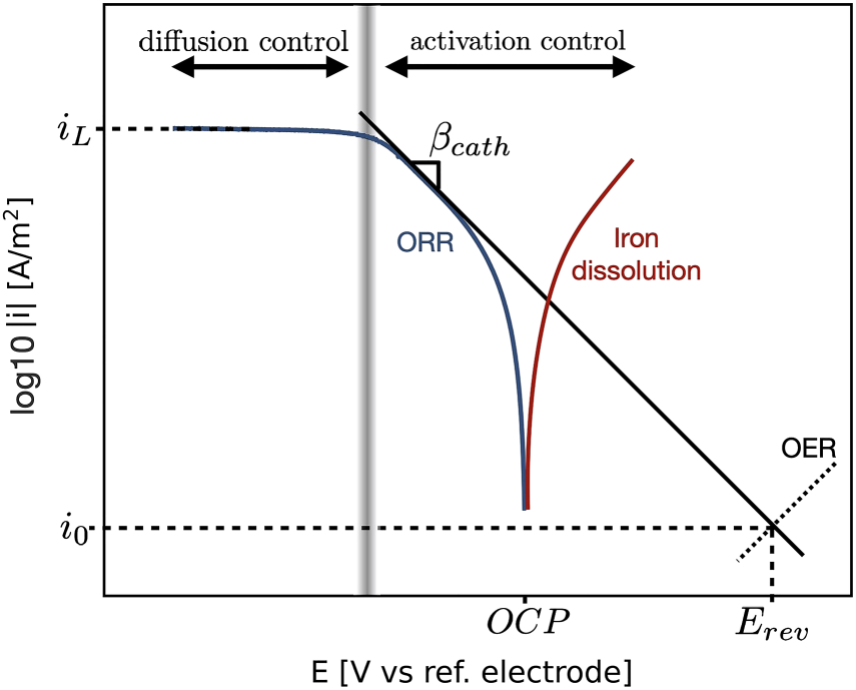
Example of a polarisation curve under mixed activation-diffusion control, showing the diffusion limiting current density, $i_{L}$ , the Tafel slope, β , the exchange current density, $i_{0}$ and the reversible potential, $E_{rev}$ , of the cathodic branch.

If the rate of an electrochemical reaction is solely limited by the charge transfer at the steel–electrolyte interface, the kinetics are activation controlled, and the relation between current density, *i*, and electrical potential, *E*, can be described by the Butler-Volmer equation,^24,25^ which is given by:^21^

(4) $$i=i_{0}*exp\left[\frac{\alpha nF(E-E_{rev})}{RT}\right]-i_{0}*exp\left[\frac{-(1-\alpha )nF(E-E_{rev})}{RT}\right]$$

where $i_{0}$ is the exchange current density, $E_{rev}$ the reversible potential and α the charge transfer coefficient of the reaction under question. *F* is the Faraday constant, *R* the gas constant, *T* the temperature and *n* the number of electrons transferred. At large over-potentials, the relation between *E* and *i* approaches linearity in a semi-logarithmic plot and is referred to as the Tafel equation.^26^ The slopes of this linear relation are called the anodic Tafel slope, $\beta_{an}$ , for the anodic branch and the cathodic Tafel slope, $\beta_{cath}$ , for the cathodic branch (Figure 1). If the charge transfer coefficient is known for a certain reaction, the associated Tafel slopes could be theoretically obtained with $\beta_{an}=\frac{2.303RT}{\alpha nF}$ and $\beta_{cath}=-\frac{2.303RT}{(1-\alpha )nF}$ .

In the case of the ORR, the kinetics are both activation and diffusion controlled. At higher over-potentials, the diffusion of oxygen to the metal surface becomes the rate-determining step, and the current-potential curve reaches a plateau, given by the limiting current density, $i_{L}$ (Figure 1). In a rotating disk electrode (RDE) setup, $i_{L}$ is linearly related to the square root of the angular frequency, ω , of the RDE,^27^ given by the Levich equation^21^:

(5) $$|i_{L}|=0.62nFD^{\frac{2}{3}}\omega^{\frac{1}{2}}v^{-\frac{1}{6}}C^{o}$$

here, *D* is the diffusion coefficient and $C^{o}$ the bulk concentration of dissolved oxygen. *v* is the kinematic viscosity of the electrolyte.

The reversible potential, $E_{rev}$ is determined by the Nernst equation.^21^ Which, at room temperature (25°C) and air pressure (1 atm), can be rewritten as:

(6) $$E_{rev}=-0.059*pH\;\mathrm{for}\;\mathrm{the}\;\mathrm{HER}$$

(7) $$E_{rev}=1.23-0.059*pH\;\mathrm{for}\;\mathrm{the}\;\mathrm{ORR}$$

The other kinetic parameters, the exchange current density, $i_{0}$ and the Tafel slopes, β , are often determined from measured current–potential curves, so called polarisation curves, as is visualised for the cathodic branch in Figure 1.

The measured value for the Tafel slope of the HER is often near the theoretical value of −0.12 V/dec, which is obtained with α = 0.5 and *n* = 1, assuming that the rate determining step is the Volmer reaction.^21–23^ On iron and steel, Tafel slopes were observed around −0.20 V/dec and even more negative.^28–31^ Radhakrishnamurthy et al.^29^ attributed relatively high absolute Tafel slopes, around −0.19 V/dec, to the presence of an oxide film on the steel. The documentation on measured exchange current densities for the HER, $i_{0,H}$ , is more scarce. For iron in acidic solution values have been observed around 1E-2 A/m^2^.^32^ For carbon steel in neutral solution a value around 4E-2 A/m^2^ was determined,^31^ and on stainless steel in near neutral solution a value of 7E-2 A/m^2^.^29^

The ORR is generally more complex than the HER, and the Tafel slopes and exchange current densities are more difficult to measure. The variation of documented values for the Tafel slope and the exchange current density of ORR is much larger. Tafel slopes measured in neutral environment on iron and steels vary from −0.060 V/dec^33^ to values around −0.12 V/dec^34–36^ up to more negative than −0.20 V/dec.^30,35,37,38^ Large ranges of Tafel slopes related to varying measurement settings or environments have also been documented. Alexander et al.^38^ observed −0.13 to −0.18 V/dec, on stainless steel for varying rotation rates of the rotating disk electrode and chloride concentrations. Babić and Metikoš-Huković^35^ observed values ranging from −0.11 to −0.18 V/dec, depending on scan direction and pH in a range of 4 to 10. For the exchange current density, values ranging from 1E-8 to 5E-4 A/m^2^ have been observed for carbon steel in neutral solution for varying chloride concentrations and diffusion layers.^30^ Jovancicevic and Bockris^34^ obtained 1E-9 A/m^2^ for actively corroding iron and 1E-3 A/m^2^ for passive iron in neutral solution.

The large spread observed in documented Tafel slopes and exchange current densities in literature, as well as the range observed by authors in their own measurements can have several reasons. First of all, there is the uncertainty resulting from the analysis of the polarisation curve. For the ORR, the Tafel region is often masked by the plateau of the diffusion limiting domain, making it difficult to correctly and consistently determine the Tafel slope and thus the exchange current density.^20^

Second, part of the spread results from variations of the investigated metal and environment. The microstructure of the metal, affected by, for example, the heat treatment, was shown to influence the corrosion kinetics of both carbon and stainless steels.^39,40^ The surface treatment of metals also affects the observed kinetics. Bozec et al.^41^ concluded that the mechanism of oxygen reduction on stainless steel is controlled by the properties of the surface, and are therefore influenced by the surface treatment. Brown et al.^42^ showed a decrease in the cathodic Tafel slope and exchange current density of hydrogen for rougher surfaces of mild steel. The environment, for example the convection of the electrolyte, directly influences the rate of diffusion of oxygen and other species towards and away from the surface.

Third, the methodology concerning the measurement of the polarisation curve can also have a large influence on the shape of the polarisation curve. Different scan directions can lead to changes of the shape of the polarisation curve, and have been attributed to the local change of the electrolyte and/or surface condition during polarisation.^22,35,37,38,43,44^ During the measurement of the polarisation curve in a neutral environment, both the electrolyte and the condition of the steel surface is altered. The polarisation of the potential away from the open circuit potential, and thus increasing the speed of the occurring cathodic reactions, may increase the pH of the electrolyte locally at the steel surface,^34^ especially in unbuffered stagnated solutions. Furthermore, at high cathodic over-potentials, oxide films at the steel surface may be removed.^22^ The presence of an oxide film may increase the cathodic Tafel slope of the ORR from −0.12 V up to sometimes even values of −0.30 V/dec.^33,35,38,45^ Finally, the scan rate of the voltammetry sweep can also affect the measured kinetics. Zhang et al.^44^ explained the effect of the scan rate on the shape of the polarisation curve, with the charging process of the interfacial capacitance. This effect is especially significant when measuring samples that show very low current densities.

## Materials and methods

We performed multiple experiments to evaluate the influence of the measurement methodology on the shape of the polarisation curve, in terms of parameters describing the kinetics of the HER and the ORR. The influence of the scan rate, scan direction, electrolyte convection and time the sample was submerged in the electrolyte were investigated with a RDE in a borate buffer solution with pH 7.5. The use of a rotating disk electrode allowed us to control the solution convection at the sample surface, by means of the rotation speed of the electrode, as well as to create a well-defined diffusion layer.^46^

The HER kinetics were studied for both the stainless and carbon steel. The ORR kinetics on the other hand were only evaluated for stainless steel. The reason for this is that in the presence of oxygen, the high corrosion rate of carbon steel in the low resistivity neutral solution, results in a low reproducibility of the obtained polarisation curves. Formed corrosion products at the surface before measurements, affect the measured kinetics and it is therefore challenging to study the variation related to a single methodology setting.

### Experimental setup

The rotating disk electrodes consisted of either a X5CrNi18-10 stainless steel disk or an S235JR carbon steel disk. The carbon steel was shown to have a typical ferrite-pearlite microstructure (see Figure A1 in the Supplemental materials). The chemical composition of both steels is given in Table 1. The disk electrodes had a diameter of 8 mm and were embedded in an insulating holder with diameter of 20 mm. Before each individual measurement, the steel surface was ground and polished with diamond paste to 1 μm , resulting in a mirror-like surface, after which the sample was degreased with ethanol and cleaned in an ultrasound bath for 3 min. The electrolyte consisted of a 0.1 M boric acid – borax buffer solution, pH 7.5, with added NaCl to reach a chloride concentration of 0.027 M.

**Table 1.** Chemical composition (wt%) of the carbon steel in accordance with the supplier information and the measured composition (wt%) of the stainless steel using energy-dispersive X-ray spectroscopy (EDS).

Material	Fe	C	Mn	P	S	N	Cu	Cr	Ni	Si
Carbon steel	≥	≤	≤	≤	≤	≤	≤	-	-	-
Stainless steel	73	*	*	*	*	*	-	18	8.0	0.58

*Not included in the EDS analysis.

**Figure 2.**
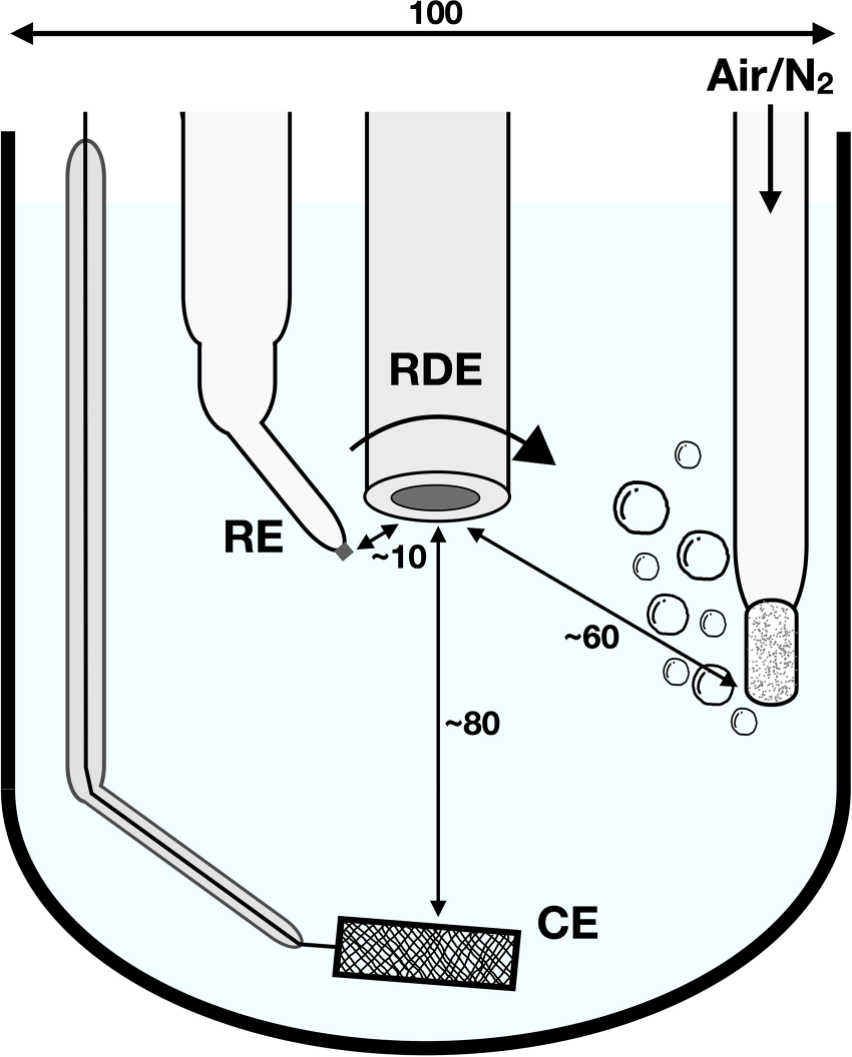
Schematic overview of the three-electrode setup, with positions of the reference electrode (RE), counter electrode (CE) and the working electrode, the rotating disk electrode (RDE). Indicated lengths between the electrodes and the dimensions of the beaker are given in mm.

The polarisation curves were obtained with cyclic sweep voltammetry (CSV) in a three-electrode setup (Figure 2). The Ag/AgCl/Sat.KCl reference electrode, was positioned next to the RDE, close to the metal surface, in a manner that it does not affect the electrolyte flow at the sample surface. A higher grade stainless steel, with a surface of approximately twice the sample surface, acted as counter electrode and was located under the RDE, at the bottom of the electrochemical cell. Depending on the studied kinetics, ORR or HER, the electrochemical cell was open and aerated by bubbling pressurised air, or closed and bubbled with N_2_ gas to minimise the concentration of oxygen in the solution, respectively. The solution was bubbled for a few minutes before the sample was submerged. For almost all experiments this meant that the solution was bubbled around more than 30 min, before the polarisation curve was measured. In the case of bubbling with N_2_ gas, this would lead to a sufficiently low dissolved oxygen concentration of smaller than 0.30 ppm.^47^ Due to the bubbling with compressed N_2_ gas, the temperature in the solutions of the HER experiments (16 °C) was lower than for the ORR experiments (room temperature, around 20 °C).

Table 2 gives an overview of the varied experimental methodologies to either study the influence of the rotation rate of the RDE, the scan rate of the CSV, or the submerge time of the sample in the electrolyte before measuring the polarisation curve. Before each experiment the working electrode was polarised for 5 min at −1.5 V versus Ag/AgCl/Sat.KCl, to remove any formed surface film. After the polarisation was finished, the RDE was kept rotating in the solution for a certain ‘submerge time’ (see Table 2) and the open circuit potential (OCP) was recorded. For submerge times of 0.5 h and up, the potential reached a stable value before the start of the measurement of the polarisation curve. For a submerge time of 0 h, the polarisation curve was measured right after 5 min of cathodic polarisation.

**Table 2.** Overview of performed experiments and the set experimental parameters. Submerged refers to the time the sample was submerged in the electrolyte before the measurement of the polarisation curves.

Studied reaction kinetics	Studied steel	Studied influence	Scan direction* & range [V vs Ag/AgCl/Sat.KCL]	Scan rate [mV/s]	Rotation rate [rpm]	Submerge time [h]	Experiment denomination
Hydrogen evolution	Stainless & carbon	Scan rate	−1.5 < -> OCP	0.151.0	1200	0.5	HER-sr
		Rotation rate	−1.5 < -> OCP	0.5	300–1800	0.5	HER-rr
Oxygen reduction	Stainless	Scan rate	−1.5 < -> OCP	0.151.0	1200	0.5	ORR-sr
		Rotation rate	−1.5 < -> OCP	0.5	600–1500	0.5	ORR-rr
		Submerge time	OCP < -> −1.5	0.5	1200	0–64	ORR-st

*Either the scan starts at −1.5 V versus Ag/AgCl/Sat.KCl and moves up towards and reversing at the open circuit potential, OCP, (−1.5 < -> OCP), or it starts at OCP and reverses at −1.5 V versus Ag/AgCl/Sat.KCl (OCP < –> −1.5).

To study the influence of the scan direction, the CSV either started at −1.5 V versus Ag/AgCl/Sat.KCl, initially measuring in a upwards scan direction up to the OCP measured before the CSV, followed by a scan back down to −1.5 V versus Ag/AgCl/Sat.KCl, or the other way round, starting at OCP and reversing at −1.5 V versus Ag/AgCl/Sat.KCl. The scan rate of the CSV and the rotation rate of the RDE were adjusted depending on the performed measurement (see Table 2). To correct for the IR-drop in the three-electrode setup, the solution resistance was determined using electrical impedance spectroscopy (EIS) after the cathodic polarisation and submerge time, directly before performing the CSV. EIS was performed in a frequency range of 1E + 5 to 1E + 3 Hz with an amplitude of 10 mV. The resistance was found from the impedance at minimum phase and was around 180 Ω . Each measurement was generally repeated three times.

### Evaluation of polarisation curves

To analyse the measured polarisation curves and to evaluate the Tafel slopes and exchange current densities, the python library PolCurveFit was applied.^20^ This library was specifically developed to fit a theoretical curve, derived from the Butler-Volmer equation (Equation 4), to the measured data, assuming the measured currents are either purely activation controlled, or mixed activation-diffusion controlled. More details can be found in earlier work.^20^

**Figure 3.**
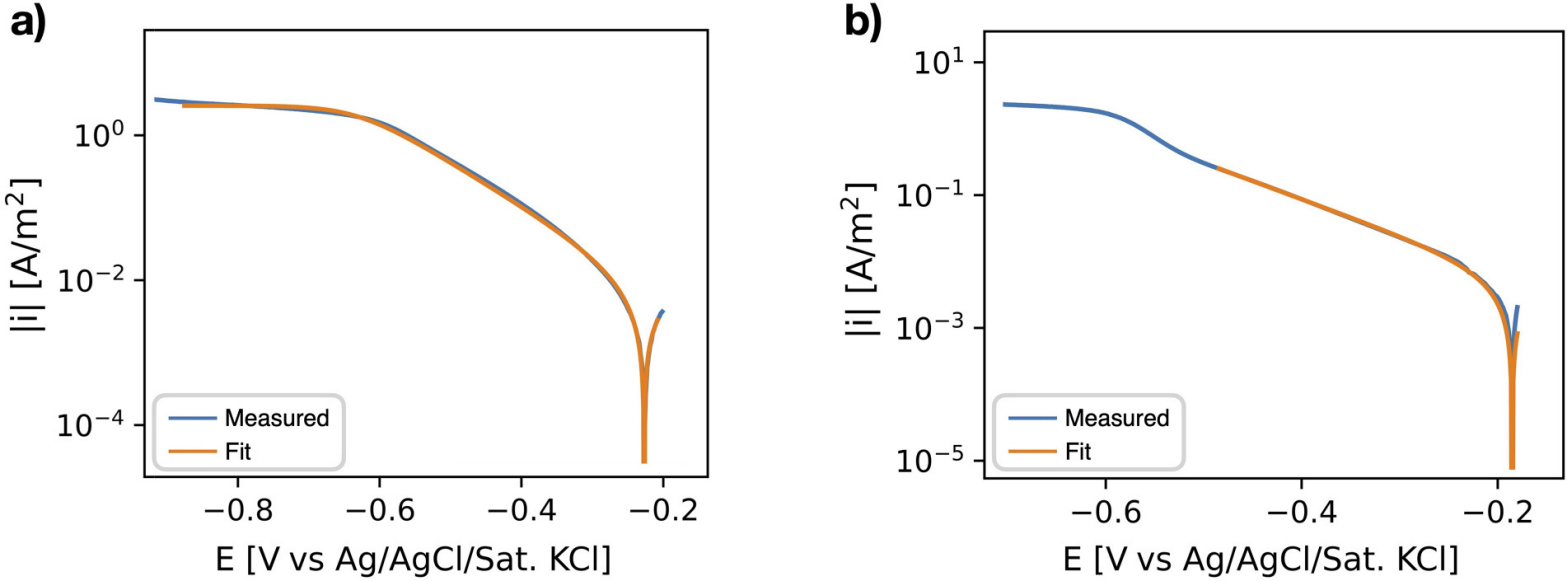
Examples of the analysis of the polarisation curves (measured for stainless steel, with a scan rate of 0.5 mV/s), using the python library PolCurveFit.^20^ (a) The fitting of a theoretical curve describing mixed activation and diffusion controlled kinetics. (b) The fitting of a theoretical curve describing purely activation controlled kinetics, down to the first change in slope.

The evaluation of the polarisation curves measured in the de-aerated electrolyte, aiming to study the HER kinetics, was performed assuming purely activation controlled kinetics. The fitted range included data starting at −200 mV versus OCP up to +50 mV versus OCP, which includes a cathodic Tafel region of almost two decades of current. The evaluation of the polarisation curves studying the ORR kinetics on stainless steel required a more differentiated approach. Two examples are shown in Figure 3. If the curve showed a clear Tafel region with a transition to a plateau describing the diffusion limiting domain, a curve describing mixed activation-diffusion control was fitted to the data (Figure 3(a)). However, if the activation controlled domain showed multiple slopes, only the data up to the first change in slope was fitted, using the ‘activation-controlled fit’ of the python library (Figure 3(b)). The exchange current densities were determined from the fitted curves, by extrapolation of the Tafel slopes to the reversible potentials (Figure 1). Assuming a fixed pH of 7.5, which is a reasonable assumption as a buffer was used here, a reversible potential of −0.44 V versus the standard hydrogen electrode (SHE) for the HER and a reversible potential of 0.79 V versus SHE for the ORR, were used (see Equations 6 and 7).

### Light reflectance spectroscopy

**Figure 4.**
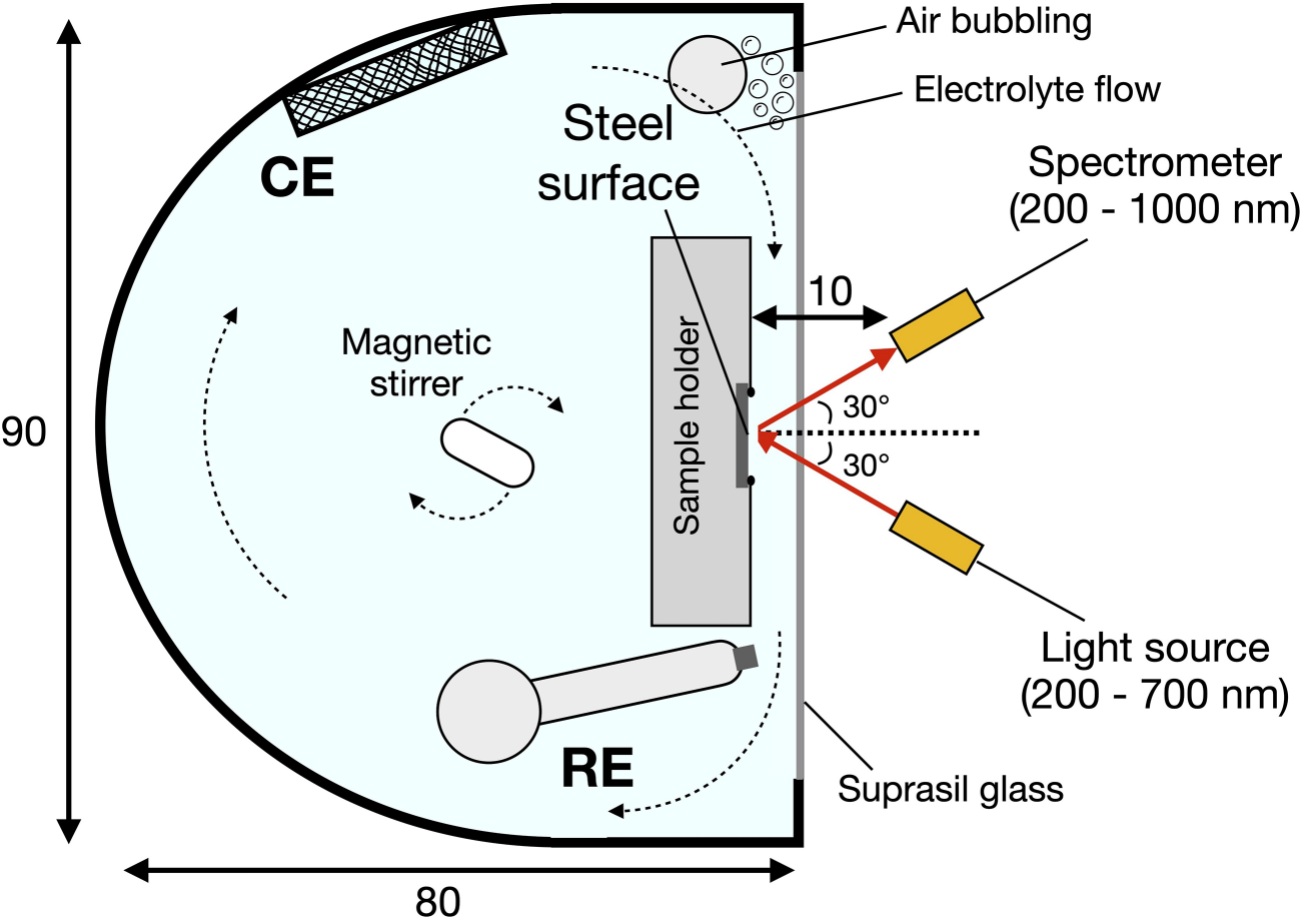
Schematic top view of the experimental setup for the reflectance spectroscopy experiment, indicating the sample, the reference electrode (RE) and the counter electrode (CE). Indicated dimensions are in mm.

During the experiments to measure the ORR kinetics on the stainless steel, an oxide film will be present, depending on the studied settings. Especially for the submerge time experiments (ORR-st, see Table 2), the stainless steel was kept in solution for long durations of time, resulting in the growth of the oxide film. To visualise this growth, during the submerge time and during the measurement of polarisation curves, we used reflectance spectroscopy in an adapted experimental setup, shown in Figure 4. Reflectance spectroscopy has been proven to be an useful technique for the in situ monitoring of the oxide film growth, for iron and carbon steel.^48–50^

In the current setup, the stainless steel was fixed in a three-dimensional (3D) printed ASA (Acrylic Styrene Acrylonitrile) sample holder, pressed against an O-ring, and placed in the electrochemical cell, orientating the steel surface parallel to the glass, which has a wavelength transparency between 220 and 2600 nm. Optical fibres, connected to a deuterium lamp (30 W) light source and the spectrometer (Thorlabs CCS200) were positioned 1 cm in front of the steel surface, with a reflection angle of 30 degrees. Next to the sample, an Ag/AgCl/Sat.KCl reference electrode was placed, as well as an inlet to bubble with air.

Two experiments were performed. First, to study the oxide film during the measurement of a polarisation curve, a similar procedure was applied as for the ORR-sr and ORR-rr experiments (Table 2). First the steel was polarised by the counter electrode for 5 min at −1.5 V versus Ag/AgCl/Sat.KCl, then the OCP was recorded for 30 min and finally the solution resistance was determined using EIS and the polarisation curve was obtained, starting at −1.5 V, up to 0.3 V and back down again to −1.5 V versus Ag/AgCl/Sat.KCl, with a scan rate of 0.5 mV/s. The reflectance spectrum was continuously recorded, with an integration time of 4100 ms. The solution was stirred and bubbled during the experiment.

Second, to show the grow of the oxide film as a function of time, the steel was polarised for 5 min at −1.5 V versus Ag/AgCl/Sat.KCl, after which the OCP was monitored for approximately 50 h, starting 5 min after the polarisation. The reflectance spectrum was recorded, with an integration time of 3800 ms. The solution was stirred, which led to a slight flow of electrolyte in front of the sample.

The recorded reflection spectra were converted to absorption spectra, *A*, using the following approximation:^48^

$$A(\lambda )=1-\frac{I(\lambda )}{I_{0}(\lambda )}$$

where λ is the wavelength, *I* the reflected light intensity, and $I_{0}$ the light intensity of the initial (oxide-free) steel surface. Using the Beer-Lambert equation, considering the geometry of the current setup, the film thickness, $d_{\;film}$ , can be computed:^50^

$$d_{\;film}[nm]=\frac{A(\lambda )}{K(\lambda )[cm^{-1}]}*\frac{cos(\alpha [\circ ])}{2}*10^{7}$$

where α is the reflection angle and A(λ) and K(λ) the absorption and the coefficient of absorption for a certain wavelength. Karlsson et al.^51^ studied the optical properties of metal oxides on stainless steel, including Fe_3_O_4_ and Cr_2_O_3_, which are most likely the dominant oxides of an oxide film on a X5CrNi18-10 stainless steel.^52^ Considering a good noise to signal ratio of our measured intensity spectra, and a high enough absorbance coefficient, we selected a wavelength of 360 nm to compute and monitor the oxide film growth. At this wavelength, *K* is approximately 2.8E + 5 cm^−1^ for Fe_3_O_4_ and 1.4E + 5 cm^−1^ for Cr_2_O_3_.^51^ The value for *K* for the oxide film on the stainless steel should lie between these two extremes.

## Results

In this section, selected results (plots and data) are presented. More detailed and individual plots of all measured polarisation curves and tables with the evaluated Tafel slopes and exchange current densities can be found in the Supplemental materials.

### Hydrogen evolution

**Figure 5.**
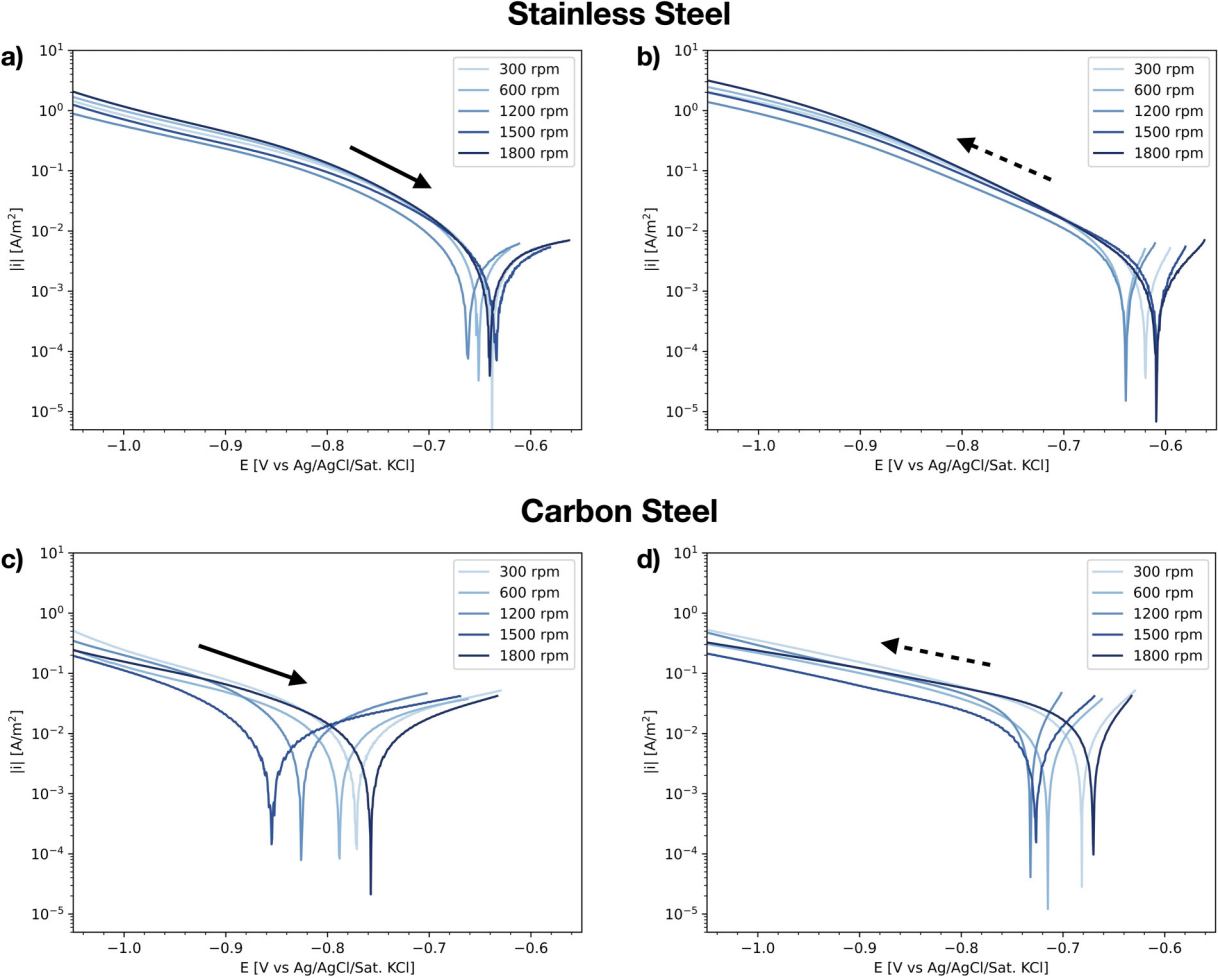
IR-drop corrected polarisation curves measured in the hydrogen evolution reaction (HER) experiments for different rotation rates (HER-rr, Table 2) in semi-logarithmic scale, representative for the repeated measurements (all curves can be found in supplemental materials B.2). (a) the upwards scan for the stainless steel, starting at −1.5 V versus Ag/AgCl/Sat.KCl up to the open circuit potential (OCP) measured before the start of cyclic voltammetric sweep. (b) The following downwards scan for the stainless steel; (c) the upwards scan for the carbon steel; and (d) the following downwards scan for the carbon steel.

To study the influence of the measurement methodology on the HER kinetics, polarisation curves were measured and evaluated for different rotation rates and scan rates, in a de-aerated electrolyte (HER-rr and HER-sr, Table 2). Measured polarisation curves for different rotation rates are compared in Figure 5 for the stainless steel and carbon steel. Figure 5(a) and (c), show the initial upwards scan, up to the OCP measured before the start of the cyclic polarisation, and Figure 5(b) and (d) show the following downwards scan. A comparison of the polarisation curves measured for different scan rates can be found in Figures B1 and B2 in the Supplemental materials. Both the curves measured on the stainless steel and carbon steel show little dependence on the scan rate and were well reproducible, the carbon steel showing a somewhat greater variation than the stainless steel.

The curves measured on stainless steel also show little dependency on the rotation rate of the RDE (Figure 5(a) and (b)). The OCP, as determined from these curves, lies between −0.61 and −0.66 V versus Ag/AgCl/Sat.KCl. This value, as well as the linear Tafel region showing solely activation controlled kinetics, suggests that the bubbling with N_2_ gas sufficiently removed the oxygen from the solution and that the cathodic branch represents the HER kinetics. The polarisation curves measured on carbon steel (Figure 5(c) and (d)) are less reproducible than stainless steel and show more variation for different rotation rates, especially for the initial upwards scan. The OCP decreases from −0.86 to −0.77 V versus Ag/AgCl/Sat.KCl with decreasing rotation rate, with the exception of the 1800 rpm curve.

**Figure 6.**
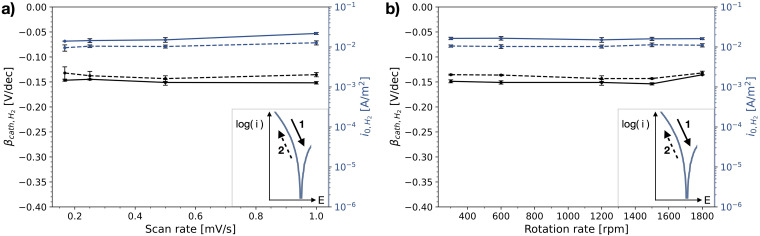
The mean and standard deviation (∼3 replicates) of the determined Tafel slopes and exchange current densities of hydrogen evolution reaction (HER) measured on stainless steel, as a function of (a) scan rate of the cyclic voltammetric sweep and (b) rotation rate of the rotating disk electrode (RDE). The solid lines represents values determined for the initial upwards scan (1), the dashed line the following downwards scan (2). All evaluated Tafel slopes and exchange current densities can be found in Tables B1 and B2 in the Supplemental materials.

That there is little variation of the polarisation curves measured on stainless steel, for different measurement methodologies, becomes even more apparent when evaluating the Tafel slope and the exchange current density of the HER. Figure 6(a) and (b) show these Tafel slopes and exchange current densities, as a function of scan rate and rotation rate, respectively, as well as for both scan directions. The determined kinetic parameters have a small standard deviation and do not show a clear dependency on the flow of the electrolyte near the metal surface, nor on the scan rate of the voltammetric scan in the investigated range.

However, there is a consistently small difference between the upwards and downwards scan. The Tafel slope of the downwards scan is for most settings less negative than that of the upwards scan, though partly within the error range. The exchange current densities are consistently lower for the downwards scan, with a small difference of 0.001 A/m^2^.

**Figure 7.**
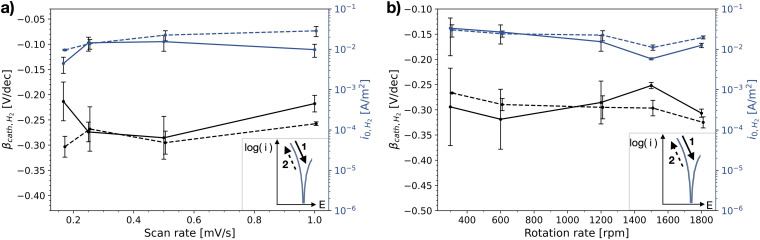
The mean and standard deviation (∼3 replicates) of the determined Tafel slopes and exchange current densities of hydrogen evolution reaction (HER) measured on the carbon steel, as a function of (a) scan rate of the cyclic voltammetric sweep and (b) rotation rate of the rotating disk electrode (RDE). The solid lines represents values determined for the initial upwards scan (1), the dashed line the following downwards scan (2). All evaluated Tafel slopes and exchange current densities can be found in Tables B3 and B4 in the Supplemental materials.

Figure 7 shows the Tafel slopes and exchange currents densities determined for the polarisation curves measured on carbon steel. The exchange current densities of HER are similar to those determined on the stainless steel. They do not show a clear dependency with rotation rate, nor with scan rate, though the individual measurements were less reproducible and a larger scatter was observed. The Tafel slopes are significantly more negative, than for those evaluated for the stainless steel, and, though no clear trend is visible, they scatter with changing scan and rotation rate.

### Oxygen reduction

**Figure 8.**
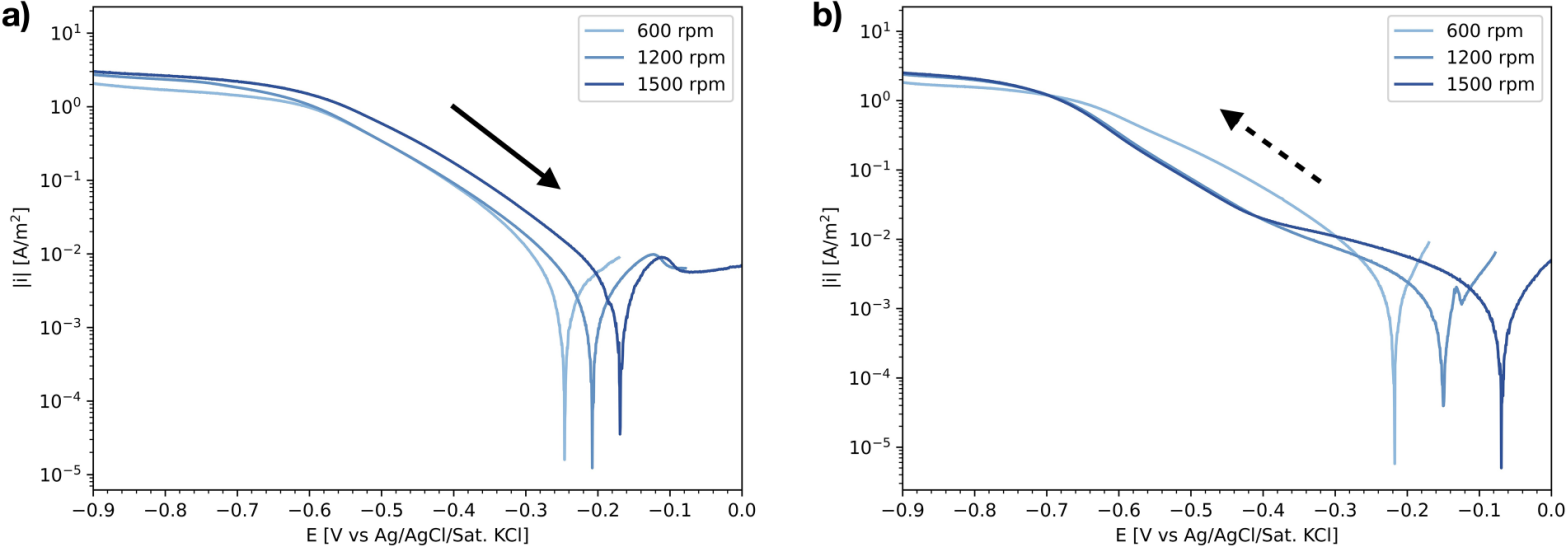
IR-drop corrected polarisation curves measured in the oxygen reduction reaction (ORR) experiments on stainless steel for different rotation rates (ORR-rr, Table 2) in semi-logarithmic scale, representative for the different repetitions (all curves can be found in Supplemental materials C.2). (a) The upwards scan, starting at −1.5 V versus Ag/AgCl/Sat.KCl up to the open circuit potential (OCP) measured before the start of cyclic voltammetric sweep. (b) The following downwards scan.

**Figure 9.**
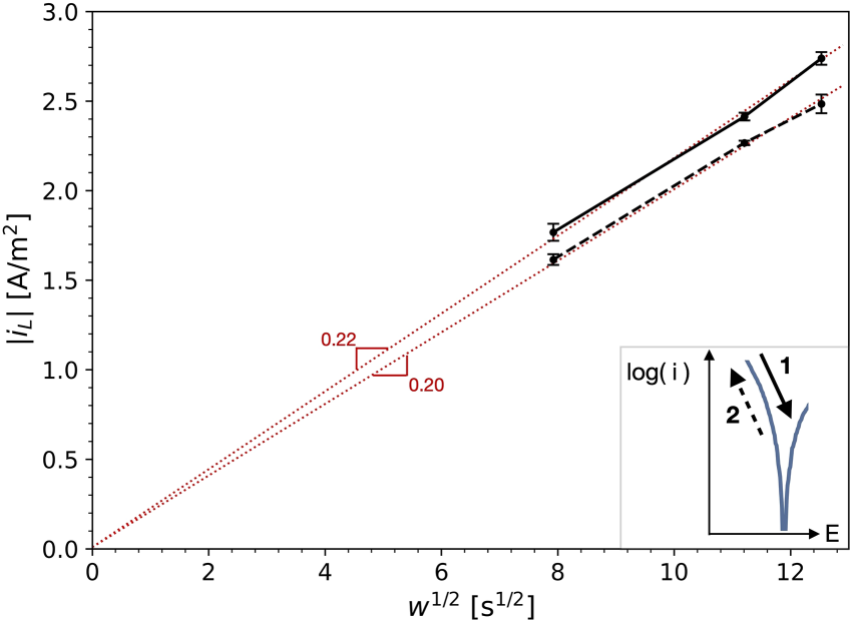
The mean and standard deviation (∼3 replicates) of the limiting current density of oxygen determined for both scan directions and at different rotation rates (Figure 8), as a function of the square root of the angular frequency, *w*. The dotted lines show the linear fit to the data through (0,0), as well as their slope.

The polarisation curves measured to evaluate the ORR kinetics were obtained in an aerated electrolyte on stainless steel (Table 2). Figure 8 shows the curves for different rotation rates and for both scan directions (ORR-rr, Table 2). A comparison of the curves for different scan rates (ORR-sr, Table 2) can be found in Figure C1 in the Supplemental materials. At lower currents, close to the OCP, the curve is mainly under activation control. At higher over-potentials the curve reaches a plateau, interpreted to be the diffusion-controlled domain and thus representing the limiting current density of oxygen, $i_{L}$ (see also Figure 1). This interpretation is confirmed by analyzing this plateau for the curves as a function of the rotation rate of the RDE. $i_{L}$ is directly related to the diffusion layer at the metal surface, and in the current setup with a well aerated electrolyte, is therefore related to the rotation speed of the rotating disk electrode, as is given by the Levich equation (Equation 5). Figure 9 shows $i_{L}$ as a function of the square root of the angular frequency *w*, for both scan directions. These curves can be fitted with a linear trend through (0,0), showing only a slight variation in the slope between the upwards and downwards scan direction. This fit matches well the Levich equation, for which the slope would be a function of parameters such as the diffusion coefficient and the kinetic viscosity.^21^

The polarisation curves for different scan rates as well as the upward scan for different rotation rates in Figure 8(a), show a relatively well reproducible shape, and little dependency on the rotation rate. There is however a shift of the OCP visible, to more positive potentials for increasing rotation rates and decreasing scan rates. In the curves for the backward scan for different rotation rates (Figure 8(b)), we see a clear change from a single linear Tafel area in the semi-logarithmic scale, to two distinct areas with increasing rotation rate.

**Figure 10.**
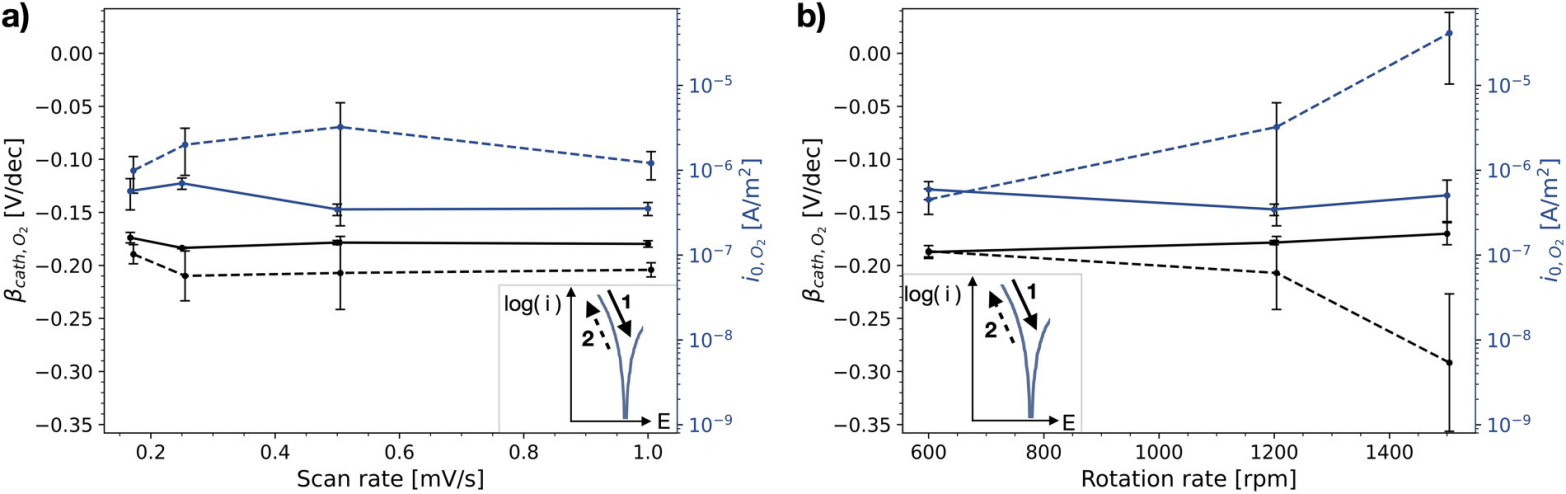
The mean and standard deviation (∼3 replicates) of the determined Tafel slopes and exchange current densities of oxygen reduction reaction (ORR) on stainless steel, as a function of (a) scan rate of the cyclic voltammetric scan and (b) rotation rate of the rotating disk electrode (RDE). The solid lines represents values determined for the initial upwards scan (1), the dashed line the following downwards scan (2). All evaluated Tafel slopes and exchange current densities can be found in Tables C1 and C2 in the Supplemental materials.

The determined Tafel slopes and exchange current densities of oxygen reduction are shown in Figure 10 for both scan directions, as a function of the scan rate and rotation rate. This figure confirms the reproducibility of the initial upwards scan, for the different repetitions, as well as the little dependency on the rotation and scan rates. For the downwards scan the curves generally showed more than one linear region, and therefore, to be consistent, the Tafel slopes and exchange current densities were determined for the first region starting from OCP (Figure 3). Not surprisingly, the kinetic parameters for the downwards scan show a large scatter, especially at larger rotation rates, and there is a clear dependency on the measurement settings. Furthermore, the offset between the two scan directions is distinct, being relatively consistent for varying scan rates and increasing for larger rotation rates.

**Figure 11.**
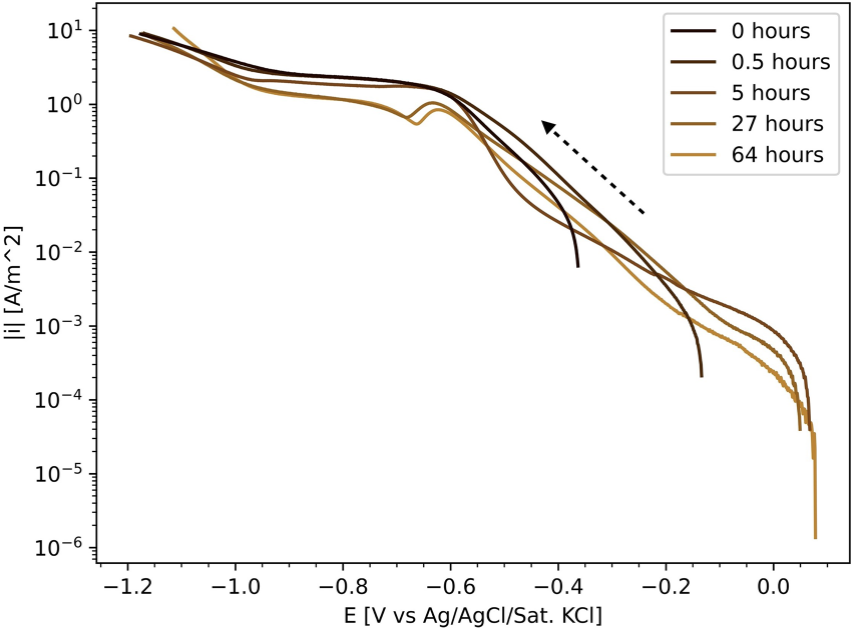
The IR-drop corrected downwards scan of polarisation curves measured in the oxygen reduction reaction (ORR) experiments on stainless steel for different submerge times (ORR-st, Table 2) in semi-logarithmic scale.

To further analyse the effect of the scan direction on the measured polarisation curves in an aerated electrolyte, experiments were performed with a reversed scanning direction (ORR-st, Table 2): starting at OCP, determined shortly before the cyclic voltammetric sweep, and scanning down until −1.5 V versus Ag/AgCl/Sat.KCl, and then back up to the OCP. Figure 11 shows the downwards scan of these curves measured for different preceding ‘submerge times’. It shows that the shape of this downwards scan heavily depends on these submerge times. The OCP increases, showing a relatively stable value for 5 h and above. The curves of 0 and 0.5 h show one distinct linear Tafel region. The higher submerge times show multiple slopes, similar to those measured at higher rotation rates (Figure 8(b)), and a bump around −0.6 V versus Ag/AgCl/Sat.KCl.

**Figure 12.**
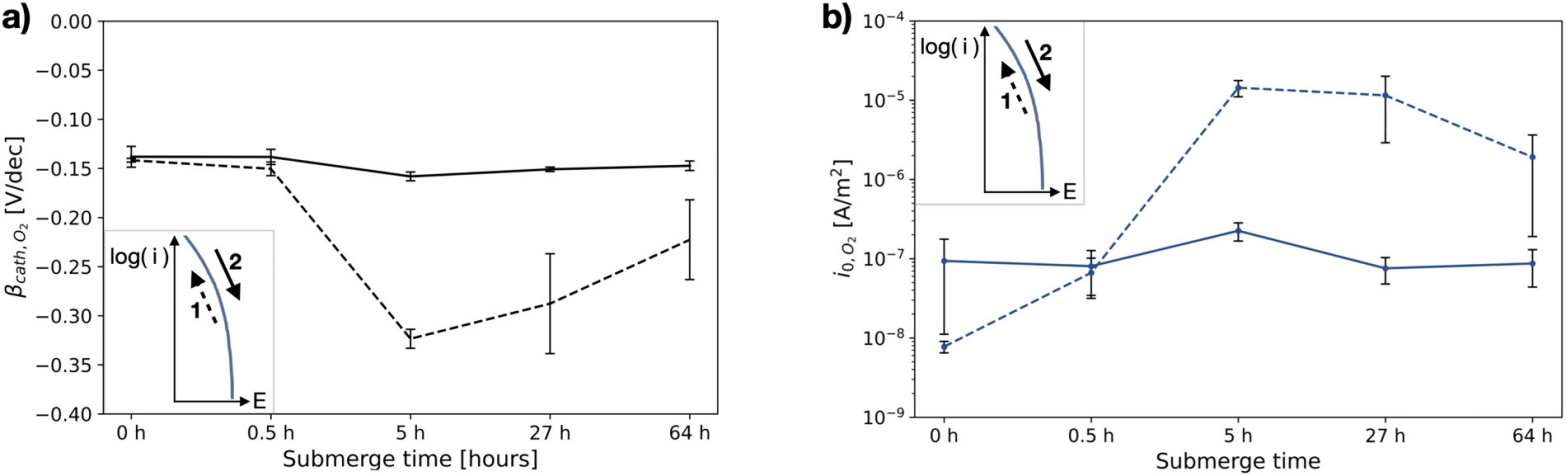
The mean and standard deviations (∼3 replicates) of (a) determined Tafel slopes and (b) exchange current densities of oxygen reduction reaction (ORR), measured on stainless steel, as a function of the submerge time of the sample between the cathodic polarisation to clean the surface, and the cyclic voltammetry scan (ORR-st, Table 2). The dashed lines represents values determined for the initial downwards scan (1), the solid lines the following upwards scan (2). All evaluated Tafel slopes and exchange current densities can be found in Tables C3 and C4 in the Supplemental materials.

The corresponding Tafel slopes and exchange current densities, for both scan directions, are shown in Figure 12. Similar to Figure 10, the parameters determined from the upwards scan are consistent, while the parameters of the downwards scan show again a dependency on the submerged time. The offset between the two scan directions is relatively small up to 0.5 h, compared to the higher submerge times. The reproducibility of the individual repeated measurements of the downwards scan decreases with increasing submerge time.

### Light reflectance spectroscopy

**Figure 13.**
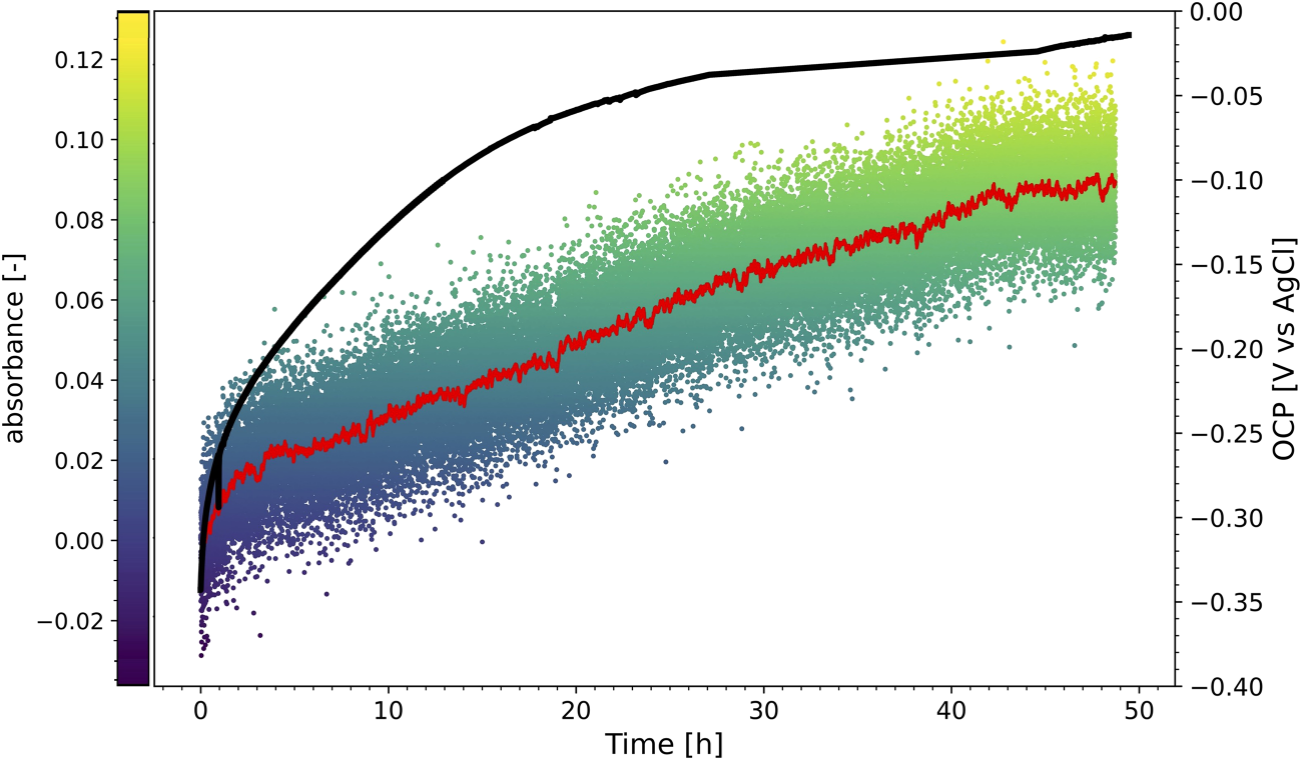
The absorbance, measured with light reflectance spectroscopy at a wavelength of 360 nm, obtained on stainless steel (scatter plot with the moving average) over time, as well as the recorded open circuit potential (OCP, solid line).

The growth of an oxide film during the submerge time before the measurement of the polarisation curves was visualised by monitoring the light reflectance over time. Figure 13 shows the computed absorbance over time, as well as the simultaneously recorded OCP. A similar behaviour as for the submerge time experiments (ORR-st, Table 2) is observed. Initially the OCP increases rapidly and after around 30 h it begins to stabilise. In the submerge time experiments that were performed in the RDE setup, this stabilisation was already reached by 5 h. While here, under less agitated conditions (Figure 4), it took longer (Figure 13). This may be explained by the fact that the RDE setup results in higher concentrations and faster transportation of oxygen, causing more rapid formation of the iron and chromium oxides. Along with the increase in OCP, the measured light absorbance increased (Figure 13). The increasing absorbance suggests the steady growth of an oxide film over this time, reaching a value of around 0.08 after 50 h, which would correspond to film thickness between 1.5 and 3.0 nm (see section ‘Light reflectance spectroscopy’ in ‘Materials and Methods').

**Figure 14.**
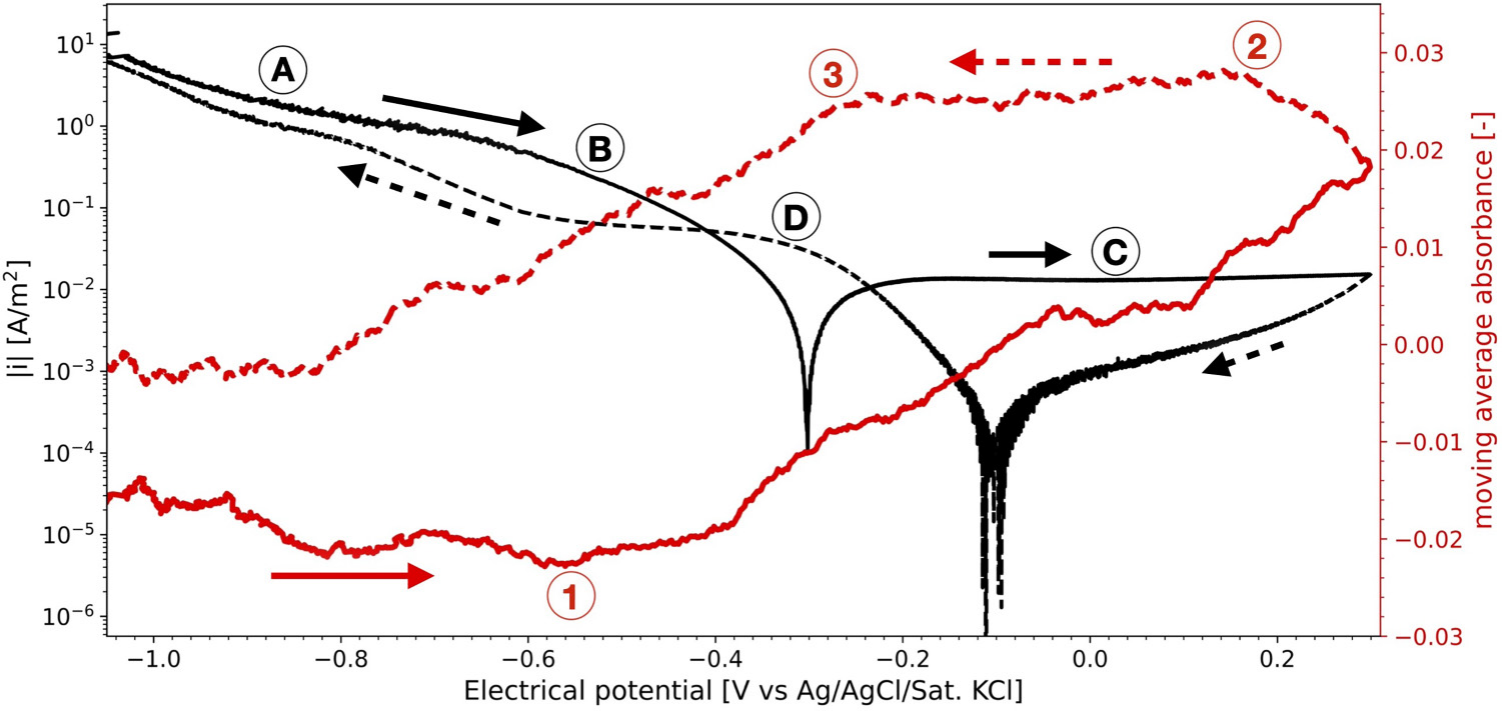
The moving average of the absorbance of the reflectance spectroscopy measurement, at a wavelength of 360 nm, recorded during the measurement of a polarisation curve on stainless steel (scan rate = 0.5 mV/s). The solid arrows indicate the initial upwards scan, the dashed arrows the subsequent downwards scan.

Light reflectance spectroscopy was also applied to study the hypothesised growth and removal of the oxide film on stainless steel during the measurement of polarisation curves in aerated solutions. Figure 14 shows the moving average of the absorbance, recorded during a polarisation curve measured with a similar procedure as the ORR-rr and ORR-sr experiments (Table 2). The polarisation curve shows a similar shape as the results of especially the ORR-rr experiments at high rotation rates (Figure 8). Starting at a negative potential, initially it reaches the oxygen diffusion-controlled domain (indicated by A in Figure 14). It is a less well-defined plateau as observed in Figure 8, as the dissolved oxygen concentration is expected to be lower in the here used setup compared to the RDE, having less agitated solution directly in front of the steel surface. Towards the OCP, the currents become solely activation controlled and a Tafel region can be observed (indicated by B). After surpassing OCP, the electrode was polarized in a typical passive region (indicated by C). On the subsequent downward scan, we observe a slope significantly different from the upward scan. Similar to the curves in Figure 8(b), we see an initial bump, indicated by D in Figure 14, before reaching the oxygen diffusion-controlled domain (A).

The absorbance shows that initially the oxide film seems to be slightly reduced. Figure 13 has shown us a steady grow of the oxide film during OCP. Therefore, we hypothesise that oxides formed at OCP during the 30 min of submerge time before the measurement of the polarisation curves, are reduced here. At around −0.6 V versus Ag/AgCl/Sat.KCl (indicated by 1 in Figure 14), the absorbance increases, marking the start of the formation of the oxide film. This potential corresponds well to the potential at which Fe_3_O_4_ becomes a stable phase.^53^ At around 0.2 V versus Ag/AgCl/Sat.KCl in the downwards scan (indicated by 2), when the anodic currents are relatively low, the formation of oxides appears to stop. Then at around −0.3 V versus Ag/AgCl/Sat.KCl (indicated by 3), at the same potentials that showed region D for the polarisation curve, the absorbance decreases, suggesting that the oxide film is being reduced again. However, although reaching a stable absorbance at around −0.8 V versus Ag/AgCl/Sat.KCl, not all of the oxide film seems to be reduced during the measurement of the cathodic branch, as the absorbance does not reach the minimum value of the initial upwards scan.

## Discussion

### Hydrogen evolution

**Figure 15.**
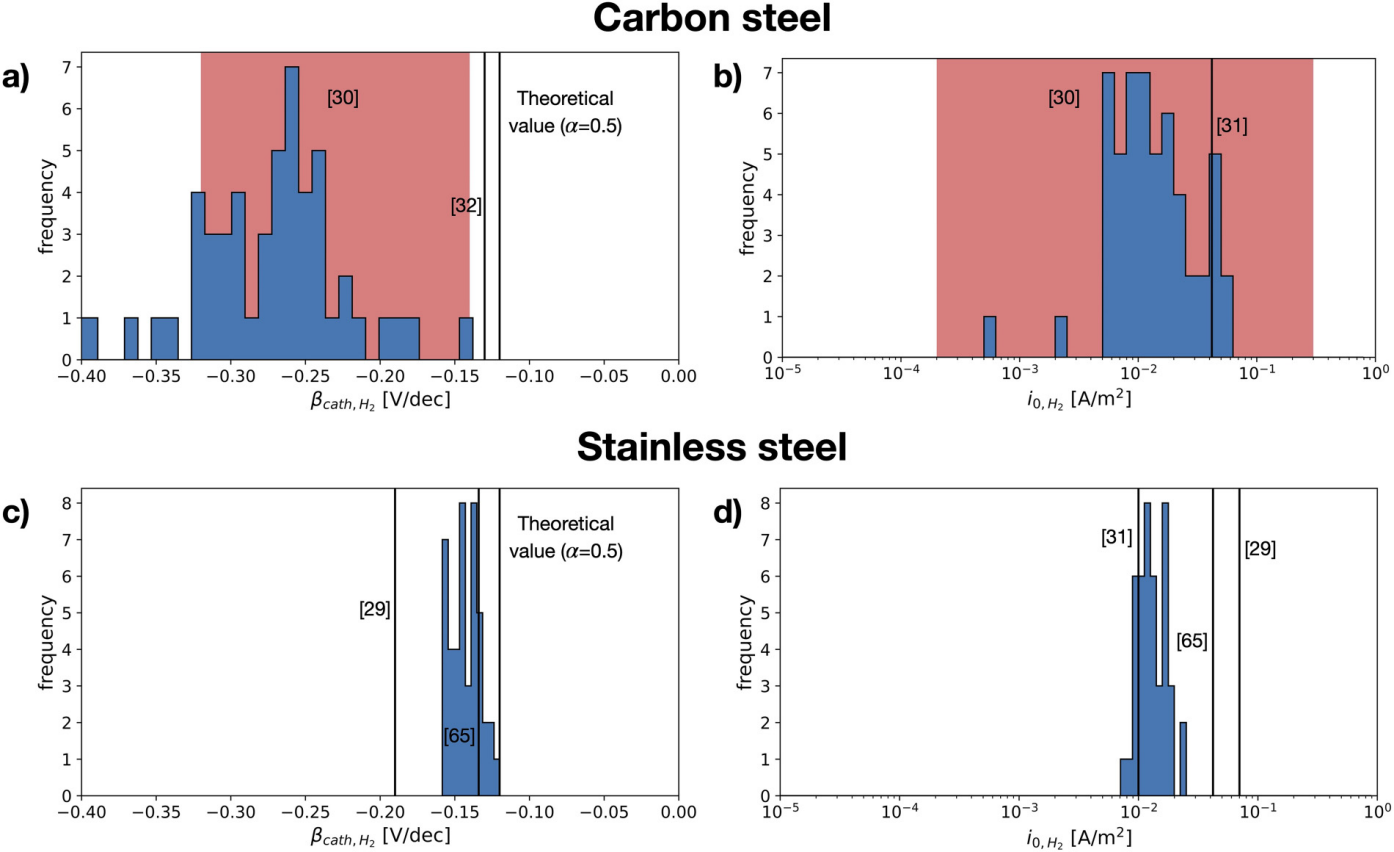
The total variation of the studied kinetic parameters of hydrogen evolution, obtained for different measurement settings: Scan rate and direction of the cyclic voltammetry and the rotation rate of the rotating disk electrode (RDE). The variations are given in histograms for (a) the cathodic Tafel slopes and (b) the exchange current density measured on carbon steel and for (c) the Tafel slope and (d) the exchange current density measured on stainless steel. Values documented in literature, measured on similar steels in neutral environments are indicated by the solid lines or with a shaded region if the authors documented a range of values, and labelled with [‘ref'].

Figure 15 illustrates the total measured variation of the studied kinetic parameters, the cathodic Tafel slopes and exchange current densities for the HER for carbon steel and stainless steel. The least variation is observed for stainless steel (Figure 15(c) and (d)). These parameters show little dependence on the studied measurement settings: the scan rate, scan direction and the rotation rates of the RDE. The Tafel slopes of hydrogen evolution are consistently determined to be around −0.13 to −0.15 V/dec. They are slightly more negative than the theoretical value of −0.12 V/dec (which assumes that the rate determining step is the Volmer reaction).^21–23^ The exchange current density of hydrogen was around 0.01–0.02 A/m^2^. These values are well in the range of earlier observed values for hydrogen evolution on stainless steel (Figure 15(d)).^29,30,54^

Tafel slopes for hydrogen reduction measured on stainless steel, more negative than the theoretical value of −0.12 V/dec, have been attributed to the presence of an oxide film.^29^ In the current experimental setup, samples were polished and after submerging in the solution, cathodically polarised, with the goal to clean the surface of any formed oxides between the polishing and the start of the measurement of the polarisation curve. However, the oxides may not have been removed completely during the cathodic polarisation. Mohammadi et al.^55^ showed that for stainless steel, some oxides remained on the steel surface, even after cathodic polarisation of 2 h at −1.5 V versus Hg/HgSO4. A recent study^56^ showed similarly that the complete reduction of surface oxides, although thermodynamically unstable, remain present under excessive cathodic polarisation for some time. These findings are in agreement with the light reflectance measurements in this work (Figure 14). The cathodic polarisation applied in the current work reached approximately −1.2 V versus Ag/AgCl/Sat.KCl (IR-drop corrected). This might not have been sufficient to reduce previously formed chromium oxides, as it could still be thermodynamically stable at this potential.^53^ We suspect that the Tafel slopes around −0.13 to −0.15 V/dec observed for the HER, can be explained by the presence of some oxides left on the steel surface.

The Tafel slope and exchange current density of hydrogen evolution measured on carbon steel, show a much larger variation than for stainless steel (Figure 15). While the exchange current densities have a similar magnitude, the Tafel slopes are significantly more negative, showing generally values around −0.24 to −0.33 V/dec. This large variation for the kinetic parameters of hydrogen evolution for carbon steel was also observed by others,^30^ although measured in a non-buffered solution.

In finding an explanation for the more negative values of HER Tafel slopes on carbon steel compared to the theoretical value of 0.12 V/dec (Section 1.1), different hypotheses proposed in the literature may be examined. For instance, for carbon steel, the measured variation by Cáceres et al.^30^ has been partly attributed to the non-Tafel-like behaviour, due to formed hydrogen gas at the surface, and the absorbed hydrogen during the measurement of the polarisation curve (see reference [22]) Another possible hypothesis may be the effect of corrosion products or oxides at the metal surface.^57^ However, this aspect was carefully examined in the present study, namely by running additional tests, increasing the time of electrochemical cleaning and decreasing the exposure time of the sample to the electrolyte before measurement. This more extensive procedure aiming at removing corrosion products and air-formed oxides had a negligible effect on the determined Tafel slope (Figure B3 in the Supplemental materials). Bao et al.^58^ found similar Tafel slopes for HER (around −0.2 V/dec) on NiMo in a near neutral buffered solution, and Lu et al.^59^ reported values around −0.4 V/dec on carbon steel in alkaline solutions. Various authors^58,60–62^ attributed this deviation from −0.12 V/dec to the contribution of concentration polarisation, arguing that in the neutral buffer solution, protons do not solely come from water dissociation and thus that the observed current densities are under mixed activation and diffusion control of the protons. As the HER current densities observed in the present study were higher for carbon steel than for stainless steel (Figure 5), this effect of concentration polarisation will be more pronounced, and might explain the larger Tafel slopes than observed on stainless steel.

An additional interesting point of discussion relates to the shape of the HER polarisation curves measured on stainless steel (Figure 5(a) and (b), Supplemental Figures B4 and B5). From these curves, we can observe two different regimes. At current densities roughly below 0.1 A/m^2^, the Tafel slopes are of the order of −0.13 to −0.15 V/dec, as reported in Figure 6; at higher current densities (and more negative potentials), however, slopes of the order of −0.25 V/dec can be observed. The slopes are comparable to the ones observed for HER on carbon steel. The current densities of carbon steel at less negative potentials are similar to the ones on stainless steel at higher negative potentials. Therefore, this may be in agreement with the hypothesis mentioned above, namely that the HER can, at least partially, be under concentration polarisation in near neutral buffer solutions when the electrode is sufficiently polarised.

### Oxygen reduction

**Figure 16.**
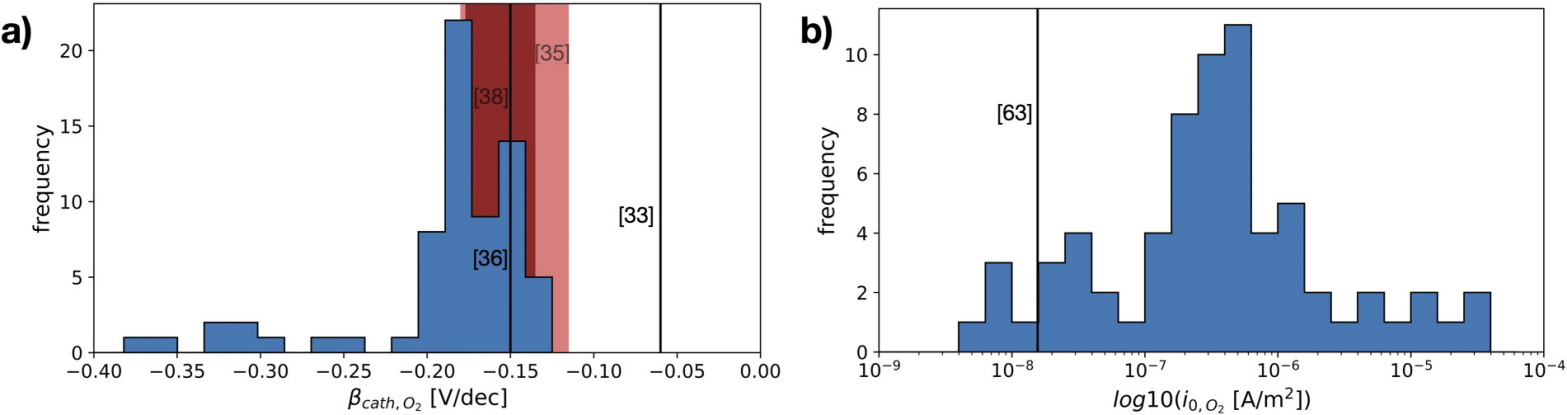
The total variation of the studied kinetic parameters for oxygen reduction, obtained for different measurement settings: Scan rate and direction of the cyclic voltammetry, the rotation rate of the rotating disk electrode (RDE), and different submerge time of the sample in solution before the measurement. The variations are given in histograms for (a) the cathodic Tafel slopes and (b) the exchange current density measured on stainless steel. Values documented in literature, measured on stainless steels in neutral environments are indicated by the solid lines or with a shaded region if the authors documented a range of values, and labelled with [‘ref’].

The studied oxygen kinetics, measured in an aerated neutral electrolyte on stainless steel, shows a high dependency on the investigated measurement settings (Figure 16), in particular the rotation speeds, submerge time and the scan direction. The Tafel slopes generally show values ranging from −0.13 to −0.21 V/dec, while the exchange current densities vary over several orders of magnitude. The measured Tafel slopes reflect well the variations of Tafel slopes of oxygen reduction on stainless steels observed in literature (Figure 16(a)).

These variations can be primarily attributed to the influence of the oxide film. The shape of the polarisation curves for the downwards scan directions at high rotation rates (Figure 8(b)) and submerge times (Figure 11), which show more than a single linear Tafel region, suggest that the kinetics are not solely defined by the reduction of oxygen on the stainless steel surface. The Tafel slope in this region, is also much more negative than when only a single linear region is visible. Earlier works observed more negative cathodic Tafel slopes due to oxide film formation.^33,35,38,45^ Recent work by Policastro et al.^63^ showed similarly shaped polarisation curves measured for stainless steel in NaCl solutions, which could be well fitted with a model describing the cathodic curve as a combination of iron oxide reduction and the ORR.

This suggested influence of the oxide film was also observed in this work, during the simultaneous measurement of light absorbance and a polarisation curve (Figure 14). After forcing the oxide film to grow, by scanning well into the anodic domain, the subsequent downwards scan of the polarisation curve shows multiple regions, while the absorbance shows that the oxide film is reduced. Thus, larger Tafel slopes in the downwards scan direction, can be explained by the reduction of the oxide film overshadowing the ORR kinetics. This becomes increasingly pronounced with increasing submerge times (Figure 11), as the oxide film has more time to grow (Figure 13). Additionally, the bump at −0.6 V versus Ag/AgCl/Sat.KCl observed for large submerge times (Figure 11), can be attributed to the reduction of iron oxides in the outer layers of the oxide film.^53,64,65^ The overshadowing of the reduction of the oxide film is also visible on the downwards scan for the experiments at different rotation rates (Figure 8), because the voltammetric scan reaches well into the potentials at which the oxide film is stable and starts to grow. The overshadowing effect becomes more pronounced with higher rotation rates, as there is faster transport of dissolved oxygen to the surface, which encourages more rapid growth of the oxide film.

The scan direction in the upwards direction showed for all experiments a good reproducibility of the kinetic parameters (Figures 6, 7, 10 and 12), independent on the submerged time, rotation rate and scan rate. Here, the oxides that for the downwards scan direction caused the overshadowing of the ORR discussed above, are expected to be already largely reduced. Arguably, this could mean that by scanning in an upwards scan direction, we initially sufficiently remove oxide films, and therefore are purely measuring the ORR kinetics.

However, our experiments show that the upwards scan direction alone is not sufficient. The absorbance monitored during the measurement of a polarisation curve (Figure 14), showed that the oxide film was not completely removed during the measurement of the cathodic branch. Moreover, at a rotation rate of 1200 rpm, scan rate of 0.5 mV/s, submerge time of 0.5 h and an upwards scan direction, we obtain a significantly lower exchange current density (around 8E-8 A/m^2^) and absolute Tafel slope (−0.14 V/dec) for the submerge time experiments (ORR-st, Table 2), than for the scan and rotation rate experiments (ORR-rr and ORR-sr, around 4E-6 A/m^2^ and −0.17 V/dec). For the former, after the half of hour of submerge time at OCP, the sample was longer cathodically polarised, as the upwards scan was preceded by a downwards scan starting at OCP. This led to more time to reduce the oxide film than for the ORR-rr and ORR-sr experiments, and thus to smaller absolute Tafel slopes. As discussed for the hydrogen evolution, others have shown that even excessive cathodic polarisation might not be sufficient to completely remove the oxide film on stainless steel^55,56^ and that the potentials applied in the current work might also not be sufficient to remove the chromium oxides.

### Implications

This work shows that the variation in recorded kinetic parameters from literature and in experiments, can, for a large part, be explained by the variation in applied electrochemical measurement methodologies, especially for the kinetic parameters of the reduction of oxygen. The presence of formed oxides or other corrosion products directly influence these kinetics. For some measured polarisation curves, this influence was clearly visible, showing multiple linear regions in the cathodic branch. However, this can only be observed if a sufficient part of the polarisation curve is measured. The reduction of the oxide film can show a Tafel region of over one decade of current (see Figure 11), and thus depending on the measured range, could be falsely identified as the reduction of oxygen.

The important question to ask, is what we actually want to measure. Corrosion engineering and research are often primarily interested in the behaviour of steel in different environments. The kinetic parameters are predominantly needed to model steel corrosion. For an accurate model, the actual kinetics occurring at the steel surface are required. This means that if the steel is passive, the modelled kinetics should take into account the reduction of the oxide film. Using documented values for the reduction of oxygen, or measuring the kinetics by scanning from negative potentials up to the OCP, might lead to the use of false values, and modelling results that do not reflect the reality. Moreover, in many applications, such as for instance the modelling of cathodic protection, the state of the steel and therefore the corrosion kinetics is expected to change over time. This change of kinetics should be considered to not over- or underestimate the corrosion rate.

Since the presented results show that Tafel slopes and exchange current densities determined from experiments may be significantly influenced by the oxidation/reduction of interfacial iron species (e.g. oxide films and rust), we also encourage future attempts to distinguish between contributions from the reaction steps involving these interfacial species and the actual anodic or cathodic reaction step occurring on the metal surface (involving charge exchange with the actual metal). Tafel slopes that are claimed to be used for the Fe/Fe^2+^ oxidation step or the ORR and HER reduction reactions, might often involve contributions from additional reactions. Stringent separation of these contributions will reduce the scatter in the literature and increase the transparency in the kinetic parameters used in different studies. Further research is needed in this regard.

Finally, even if the state of the steel is considered correctly and values for the corrosion kinetics are chosen or measured accordingly, this work shows that the measurement settings can heavily influence the obtained values, and might not represent reality. In the ideal case the Tafel slopes and exchange current densities are measured in their natural environment, in terms of the state of the steel surface and environment. However, as mentioned, for applications were the numerical modelling of corrosion is especially valuable, such as the example of corrosion in porous media, this is not feasible. Work by Duprat,^19^ who measured polarisation curves in a large amount of reinforced concrete samples, found large coefficients of variation for the anodic and cathodic Tafel slopes. The current work and the visible spread in literature, shows us that at this moment we cannot consider the kinetic parameters to be ‘known and fixed constants’ in our model. Especially the dependency of the oxygen kinetics on environmental factors and measurement methodology, increases the change of inaccurate values used for simulations. With the increasing computational power, it would be beneficial to use probabilistic modelling instead, explicitly modelling the uncertainty of the kinetic parameters, and therefore obtaining a realistic uncertainty of the modelling results.

## Conclusions

The current work showed that even in a controlled laboratory setup with a buffered electrode, a significant spread in measured kinetics can be found, and that this can be explained by variations in the measurement methodology. The following major conclusions are drawn:

1. Tafel slopes and exchange current densities for the HER measured on stainless steel were observed to be most reproducible, showing Tafel slopes around −0.13 to −0.15 V/dec and exchange current densities around 0.01 to 0.02 A/m^2^.
2. The HER kinetics studied on carbon steel showed a much larger variation. Exchange current density were found ranging from 0.004 to 0.05 A/m^2^ and Tafel slopes were significantly higher than for stainless steel (−0.24 to −0.33 V/dec), which may be explained by the contribution of concentration polarization.
3. The obtained ORR kinetics measured on stainless steel showed a clear dependence on the scan direction of the voltammetric scan, the solution convection at the steel surface and the time of exposure to the electrolyte prior to the scan. Tafel slopes were observed ranging from −0.13 to −0.21 V/dec, and exchange current densities varied over several orders of magnitude. By active light reflectance measurements during a voltammetric scan, the large variation could be attributed to the influence of an oxide film. At small over-potentials, the reduction of the oxide film can overshadow the ORR kinetics, leading to higher absolute Tafel slopes and exchange current densities. This oxide film can form during the measurement of the polarisation curve or remain from previous exposure to the electrolyte, due to insufficient electrochemical cleaning procedures. The influence of the reduction of the oxide film on the polarisation curve may lead to misinterpretation of the derived Tafel slope (erroneously taken as the Tafel slope of the ORR), depending on the measured range of the polarisation curve.
4. The obtained variation does not only give us insight on the accuracy of measured or documented kinetic parameters in literature, it also shows us that we cannot use these parameters as fixed constants in electrochemical techniques or in the numerical modelling of steel corrosion. For the last, we recommended on explicitly modelling the uncertainty of these parameters, to increase the accuracy of corrosion models.

## Supplemental Material

sj-docx-1-ces-10.1177_1478422X241227829 - Supplemental material for Tafel slopes and exchange current densities of oxygen reduction and hydrogen evolution on steel Supplemental material, sj-docx-1-ces-10.1177_1478422X241227829 for Tafel slopes and exchange current densities of oxygen reduction and hydrogen evolution on steel by M. C. van Ede and U. Angst in Corrosion Engineering, Science and Technology
