# Supplementary material for: Tafel slopes and exchange current densities of oxygen reduction and hydrogen evolution on steel
Source: Corros Eng Sci Technol. 2024 Jan 30;59(1):39–55. doi: 10.1177/1478422X241227829 (PMC13086234; doi:10.1177/1478422X241227829)
Supplement: sj-docx-1-ces-10.1177_1478422X241227829 - Supplemental material for Tafel slopes and exchange current densities of oxygen reduction and hydrogen evolution on steel [file sj-docx-1-ces-10.1177_1478422X241227829.docx]

Supplementary Materials
Tafel slopes and exchange current densities of oxygen reduction and hydrogen evolution on steel

M.C. van Ede${}^{1}$, U. Angst${}^{1}$*

${}^{1}$Institute for Building Materials, ETH Zurich, 8093, Zurich, Switzerland.

*E-mail: uangst@ethz.ch

# A. Microstructure


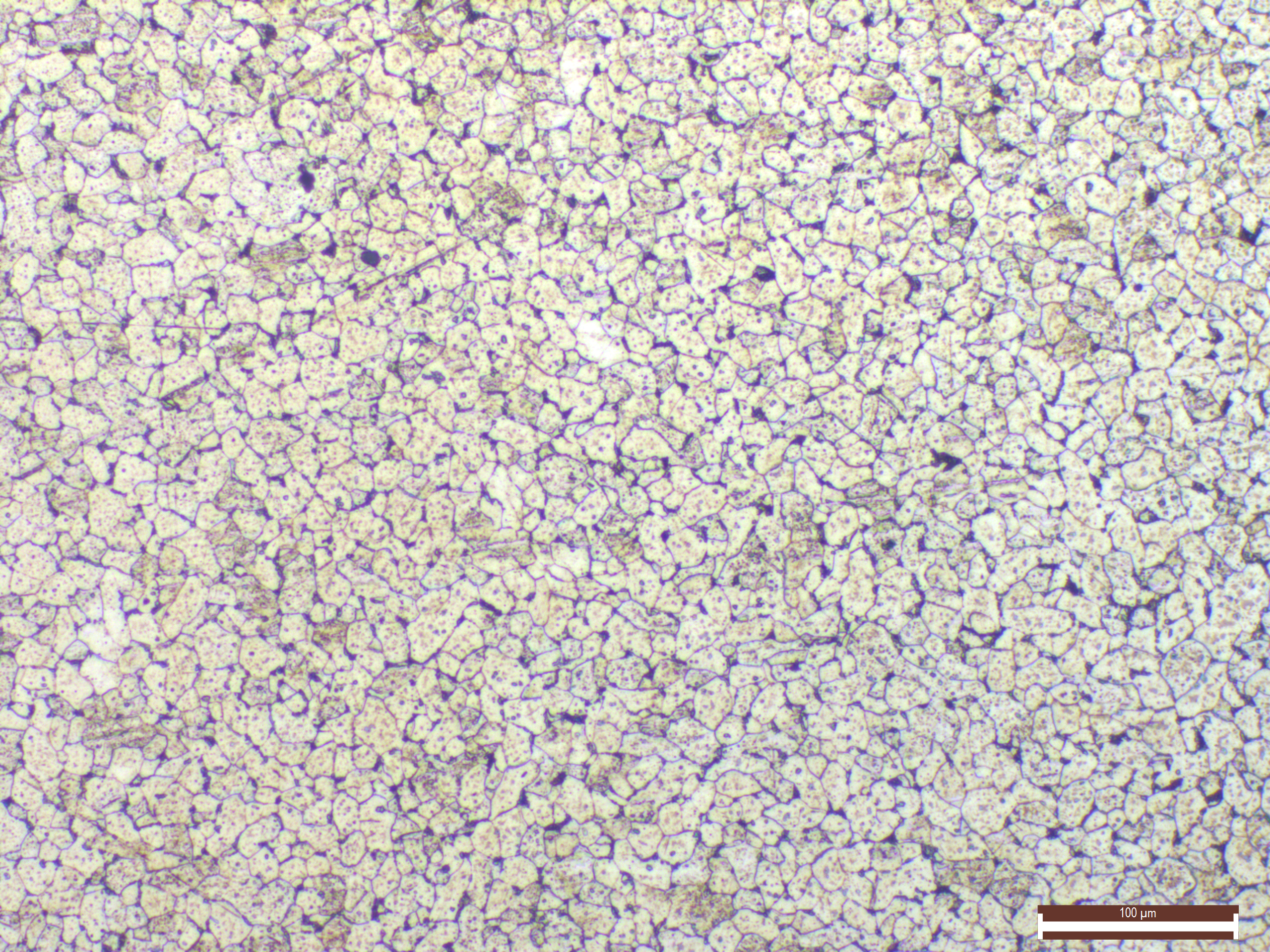


Figure A1. Microstructure of the Carbon steel sample (optical microscopy, etched with Nital), showing a typical ferrite-pearlite structure.

# B. Hydrogen evolution

## B1. Supplementary figures


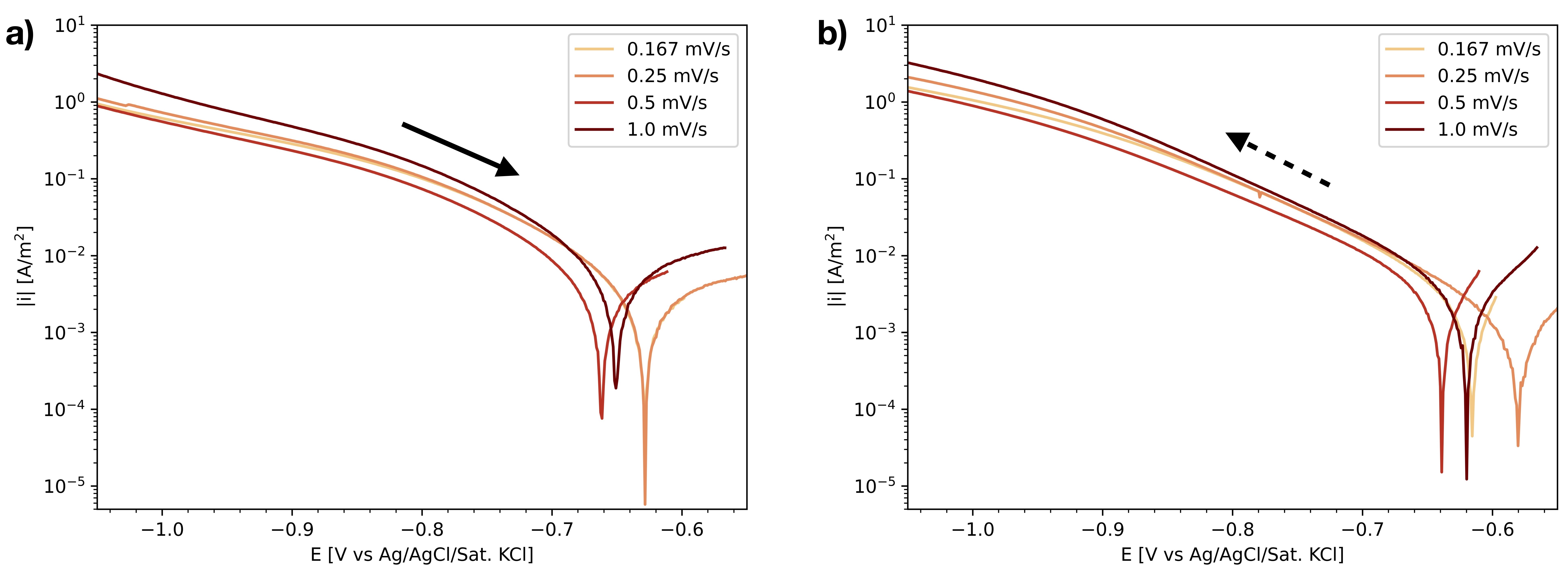


Figure B1. Measured polarization curves of stainless steel in the hydrogen evolution experiments for different scan rates (HER-sr, table 2) in semi-logarithmic scale. a) the upwards scan, starting at -1.5 V vs Ag/AgCl/Sat.KCl up to the OCP measured before the start of cyclic voltammetric scan. b) the following downwards scan.


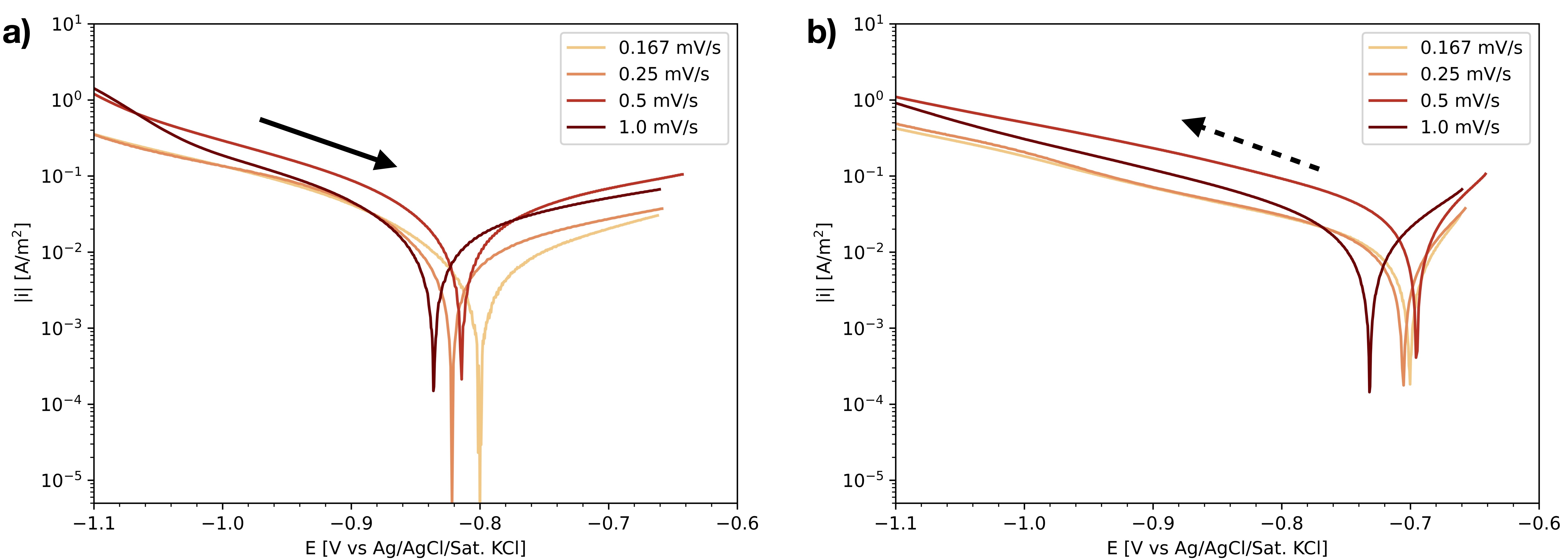


Figure B2. Measured polarization curves of carbon steel in the hydrogen evolution experiments for different scan rates (HER-sr, table 2) in semi-logarithmic scale. a) the upwards scan, starting at -1.5 V vs Ag/AgCl/Sat.KCl up to the OCP measured before the start of cyclic voltammetric scan. b) the following downwards scan.


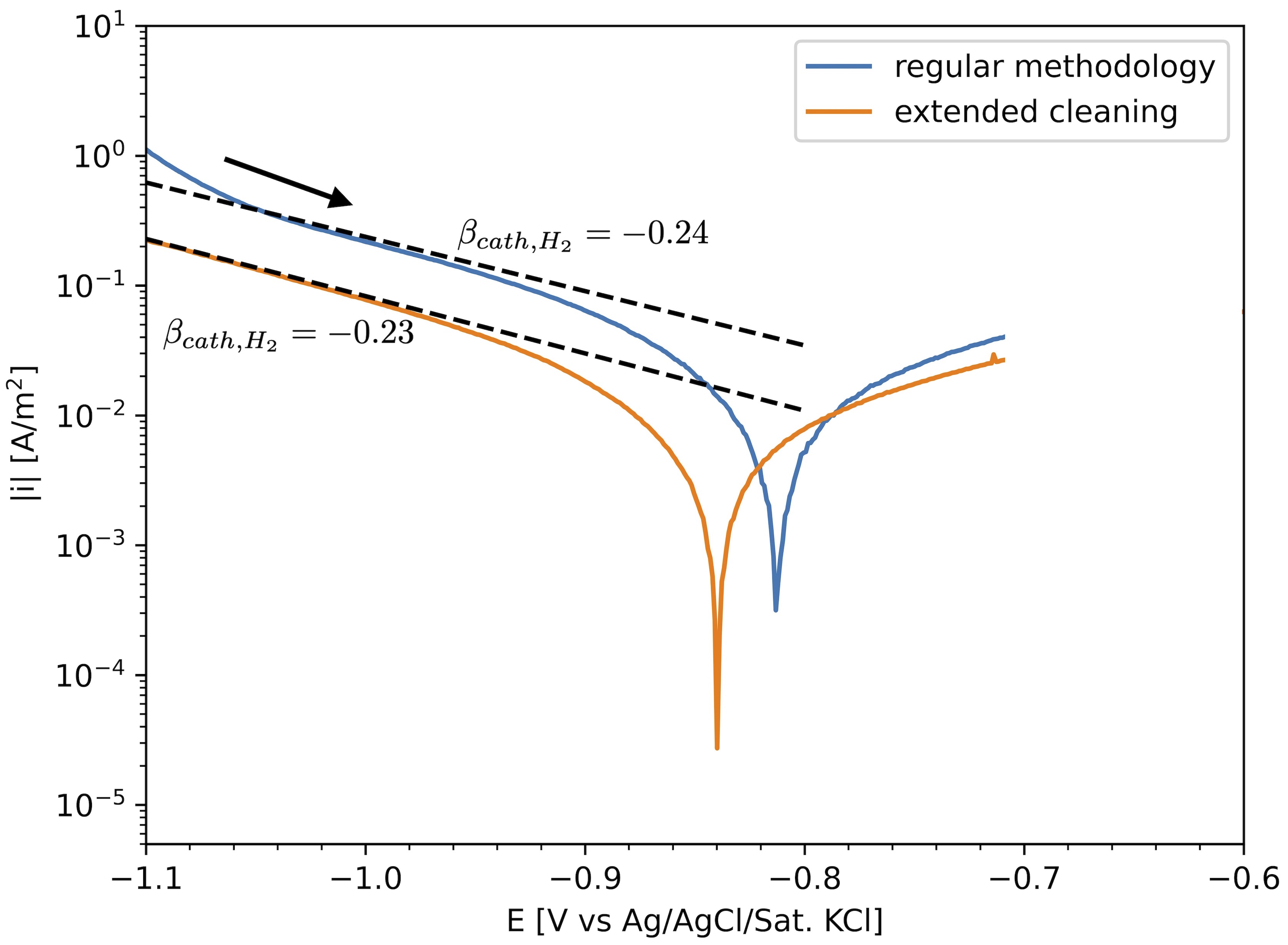


Figure B3. Comparison of measured polarization curves and Tafel slopes of different cleaning procedures, studying the HER kinetics on carbon steel. In the regular methodology, the steel was polarized for 5 min at -1.5 V vs Ag/AgCl/Sat.KCl, after which it was left exposed to the electrolyte for around 0.5 hours before the start of the measurement of the polarization curve. In the extended cleaning procedure, the steel was polarized for 15 min, after which the polarization curve was directly measured. The curves were measured in an upward scan direction, starting at -1.5 V vs Ag/AgCl/Sat.KCl up to the OCP, with a scan rate of 0.5 mV/s and at a RDE rotation rate of 1200 rpm.

## B.2 All measured polarization curves


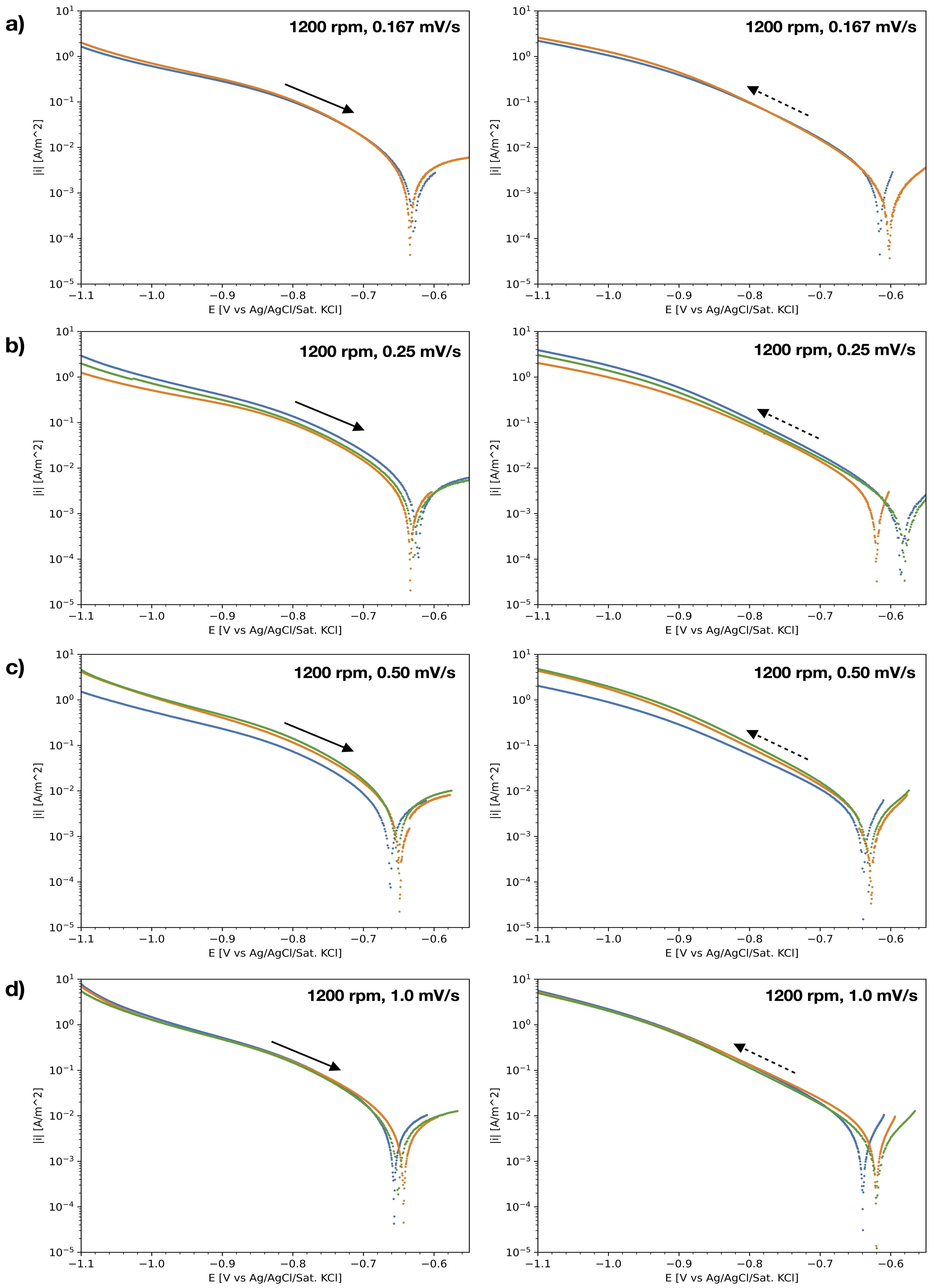


Figure B4. All IR-drop corrected polarization curves, measured with a RDE on stainless steel in a de-aerated neutral borate buffer solution, for different scan rates: a) 0.167 mV/s, b) 0.25 mV/s, c) 0.5 mV/s, d) 1.0 mV/s (HER-sr, table 2). Left) the initial upwards scan, right) the following downwards scan.


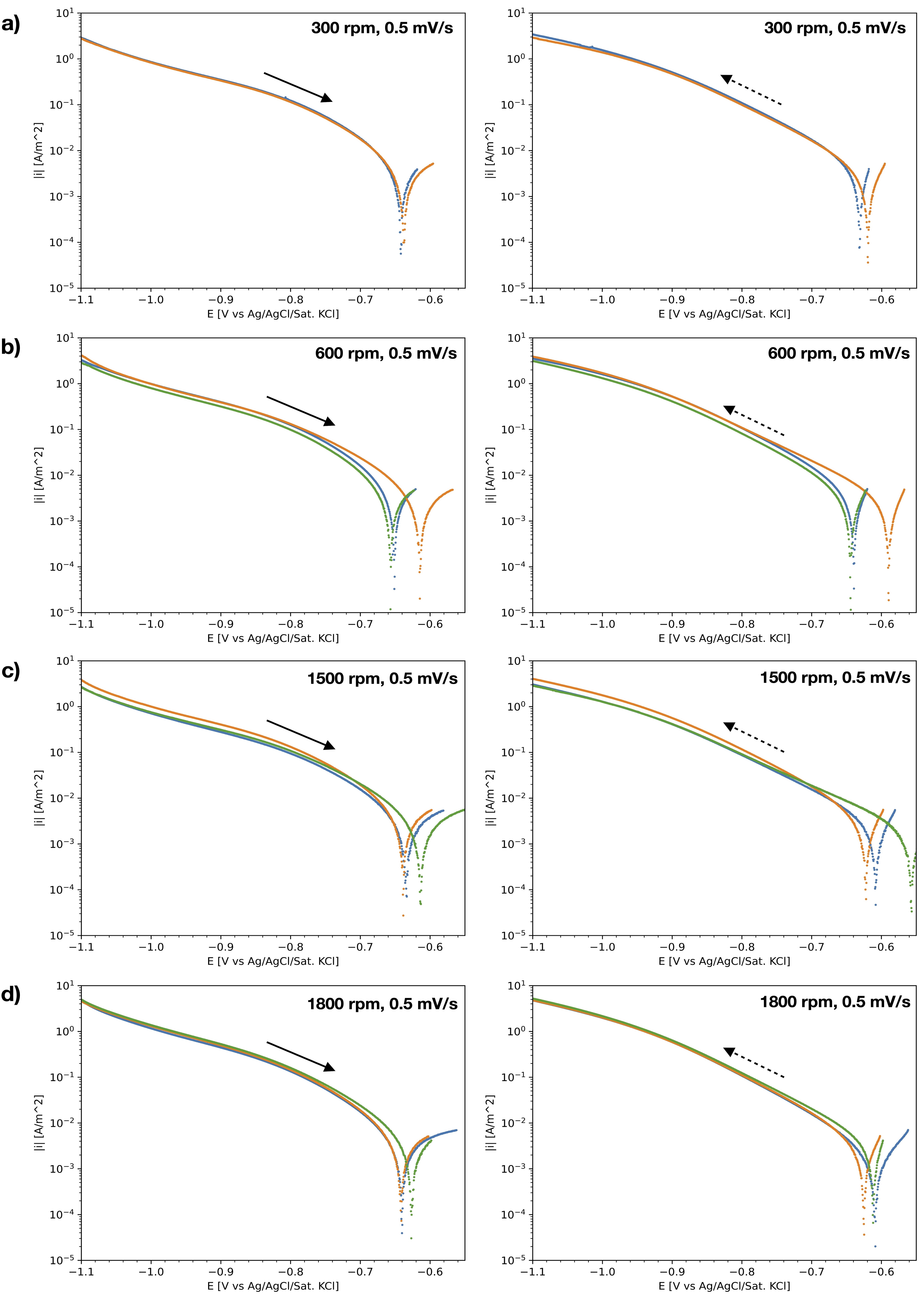


Figure B5. All IR-drop corrected polarization curves, measured with a RDE on stainless steel in a de-aerated neutral borate buffer solution, for different rotation rates: a) 300 rpm, b) 600 rpm, c) 1500 rpm, d) 1800 rpm (HER-rr, table 2). Left) the initial upwards scan, right) the following downwards scan.


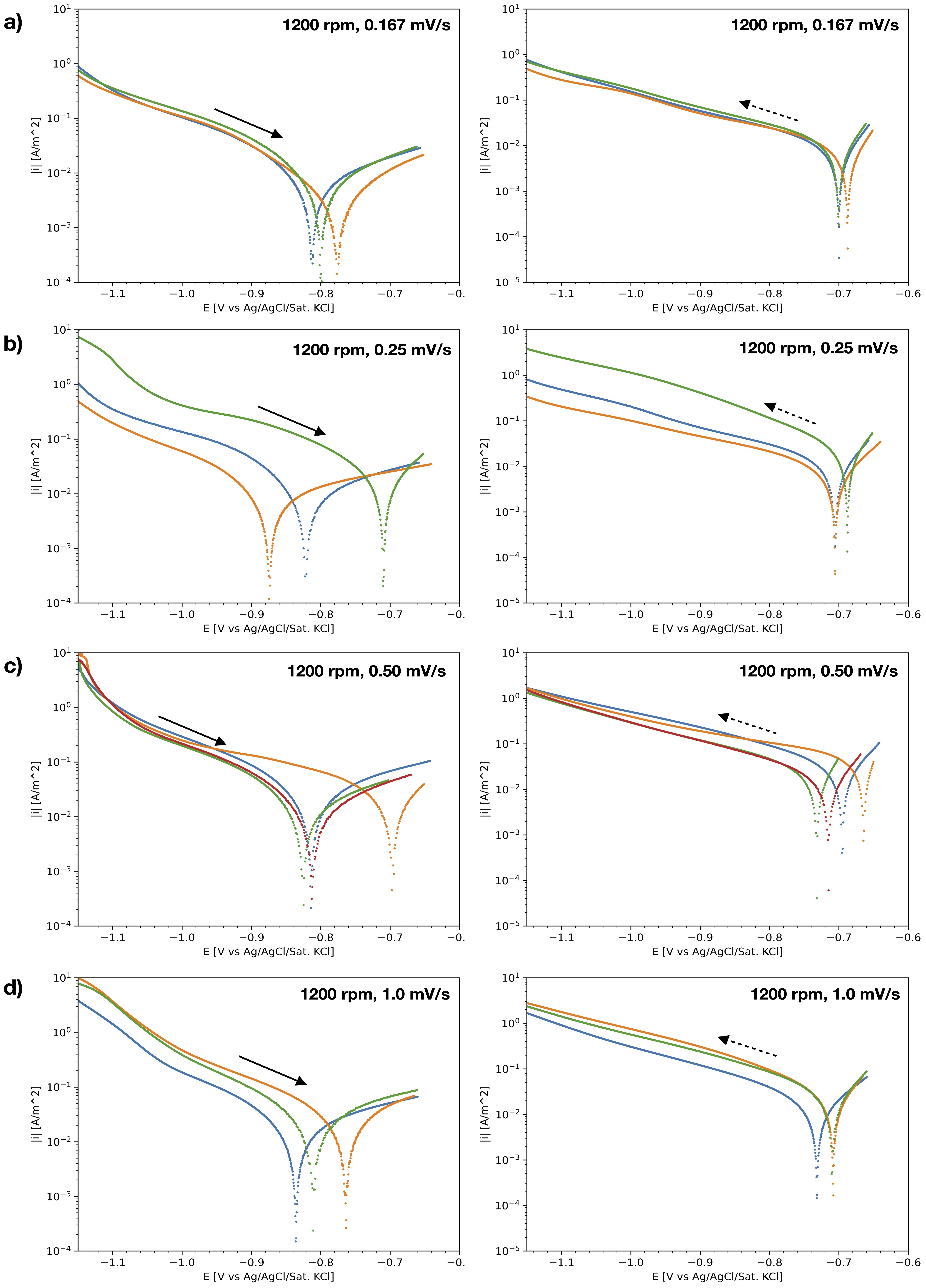


Figure B6. All IR-drop corrected polarization curves, measured with a RDE on carbon steel in a de-aerated neutral borate buffer solution, for different scan rates: a) 0.167 mV/s, b) 0.25 mV/s, c) 0.50 mV/s, d) 1.0 mV/s (HER-sr, table 2). Left) the initial upwards scan, right) the following downwards scan.


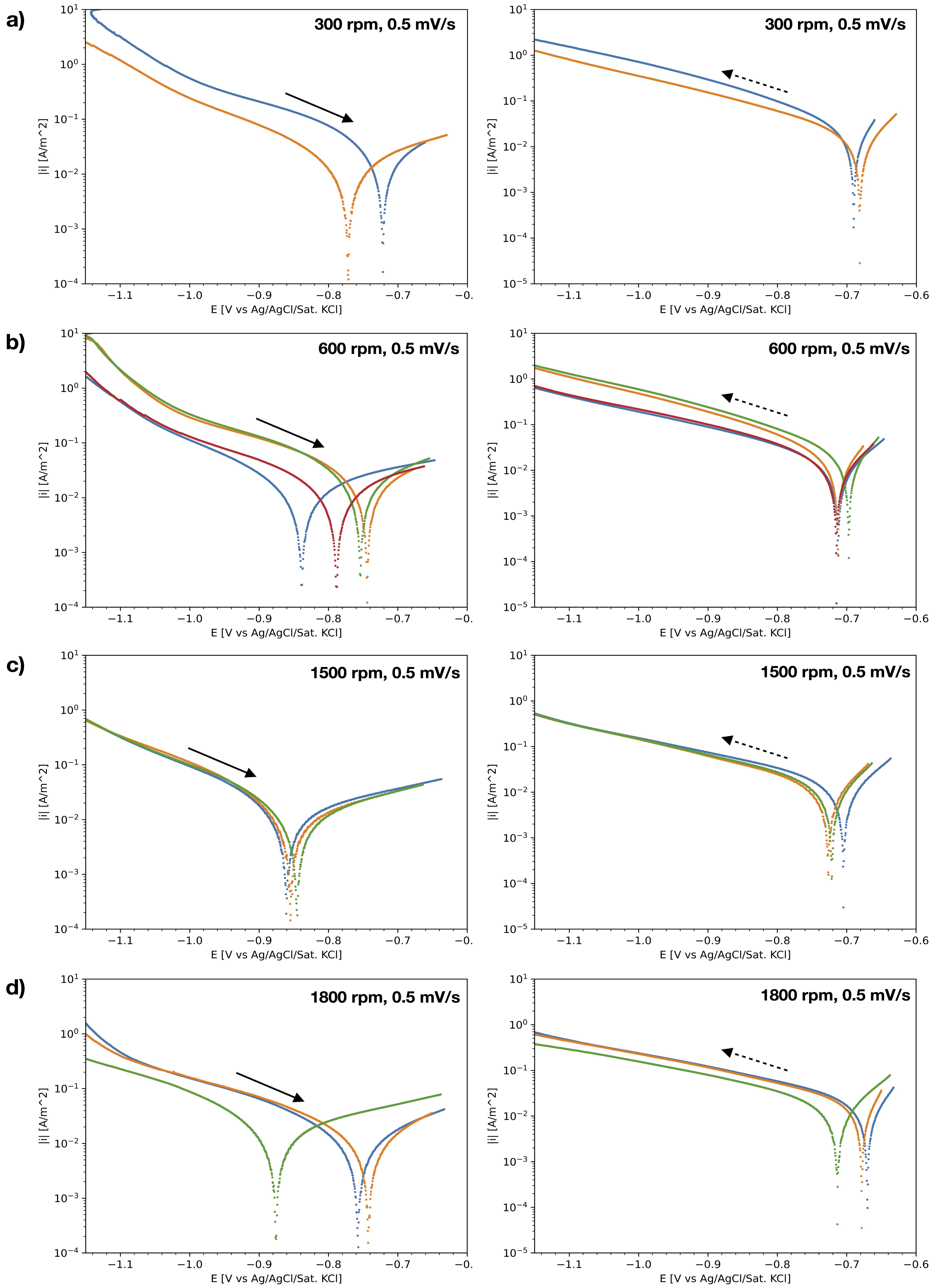


Figure B7. All IR-drop corrected polarization curves, measured on carbon steel with a RDE in a de-aerated neutral borate buffer solution, for different rotation rates: a) 300 rpm, b) 600 rpm, c) 1500 rpm, d) 1800 rpm (HER-rr, table 2). Left) the initial upwards scan, right) the following downwards scan.

## B.3 Fitted kinetic parameters

Table B1. Kinetic parameters of the hydrogen evolution reaction, obtained by fitting polarization curves measured in an upwards scan direction (-1.5 V vs Ag/AgCl/Sat.KCl –> OCP) on stainless steel in a de-aerated neutral Borate Buffer (section B.2).

*Table B2. Kinetic parameters of the hydrogen evolution reaction, obtained by fitting polarization curves measured in a downwards scan direction (-1.5 V vs Ag/AgCl/Sat.KCl –> OCP) on stainless steel in a de-aerated neutral Borate Buffer (section B.2).*

Table B3. Kinetic parameters of the hydrogen evolution reaction, obtained by fitting polarization curves measured in an upwards scan direction (-1.5 V vs Ag/AgCl/Sat.KCl –> OCP) on carbon steel in a de-aerated neutral Borate Buffer (section B.2).

Table B4. *Kinetic parameters of the hydrogen evolution reaction, obtained by fitting polarization curves measured in a downwards scan direction (-1.5 V vs Ag/AgCl/Sat.KCl –> OCP) on carbon steel in a de-aerated neutral Borate Buffer (section B.2).*

# C. Oxygen reduction

## C.1 Supplementary figures


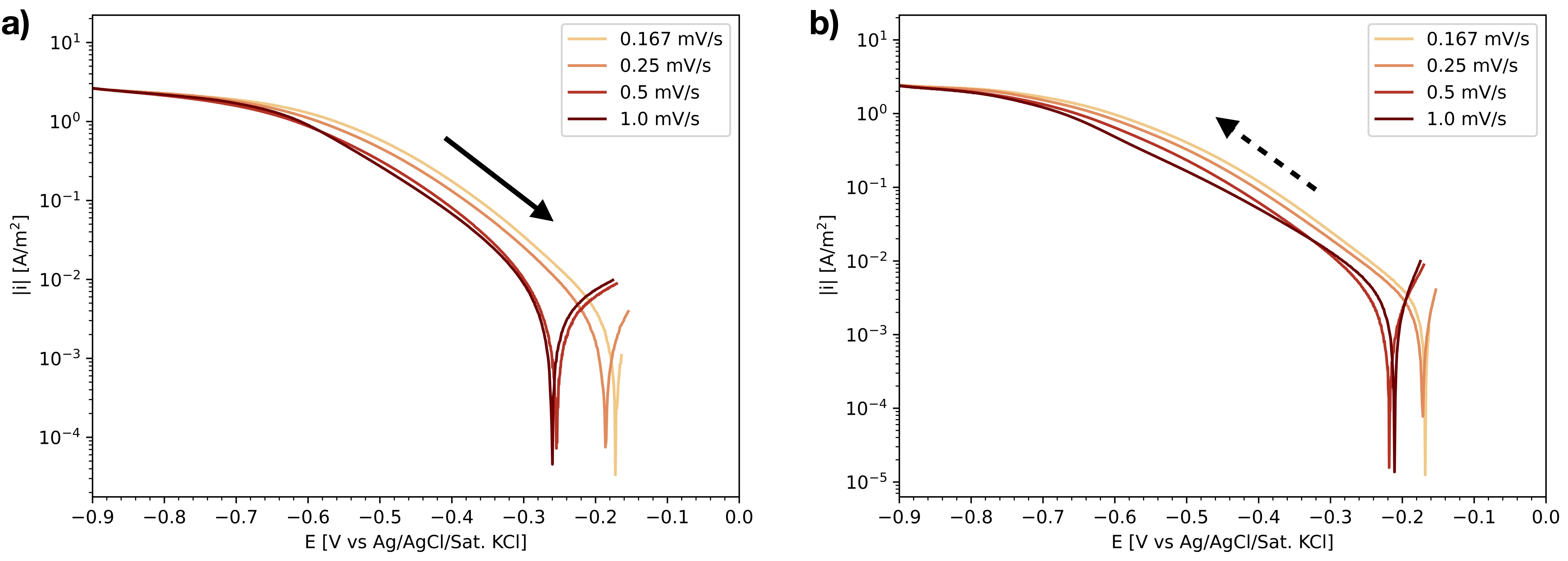


Figure C1. Measured polarization curves in the oxygen reduction experiments for different scan rates (ORR-sr, table 2) in semi-logarithmic scale. a) the upwards scan, starting at -1.5 V vs Ag/AgCl/Sat.KCl up to the OCP measured before the start of cyclic voltammetric scan. b) the following downwards scan.

## C.2 All measured polarization curves


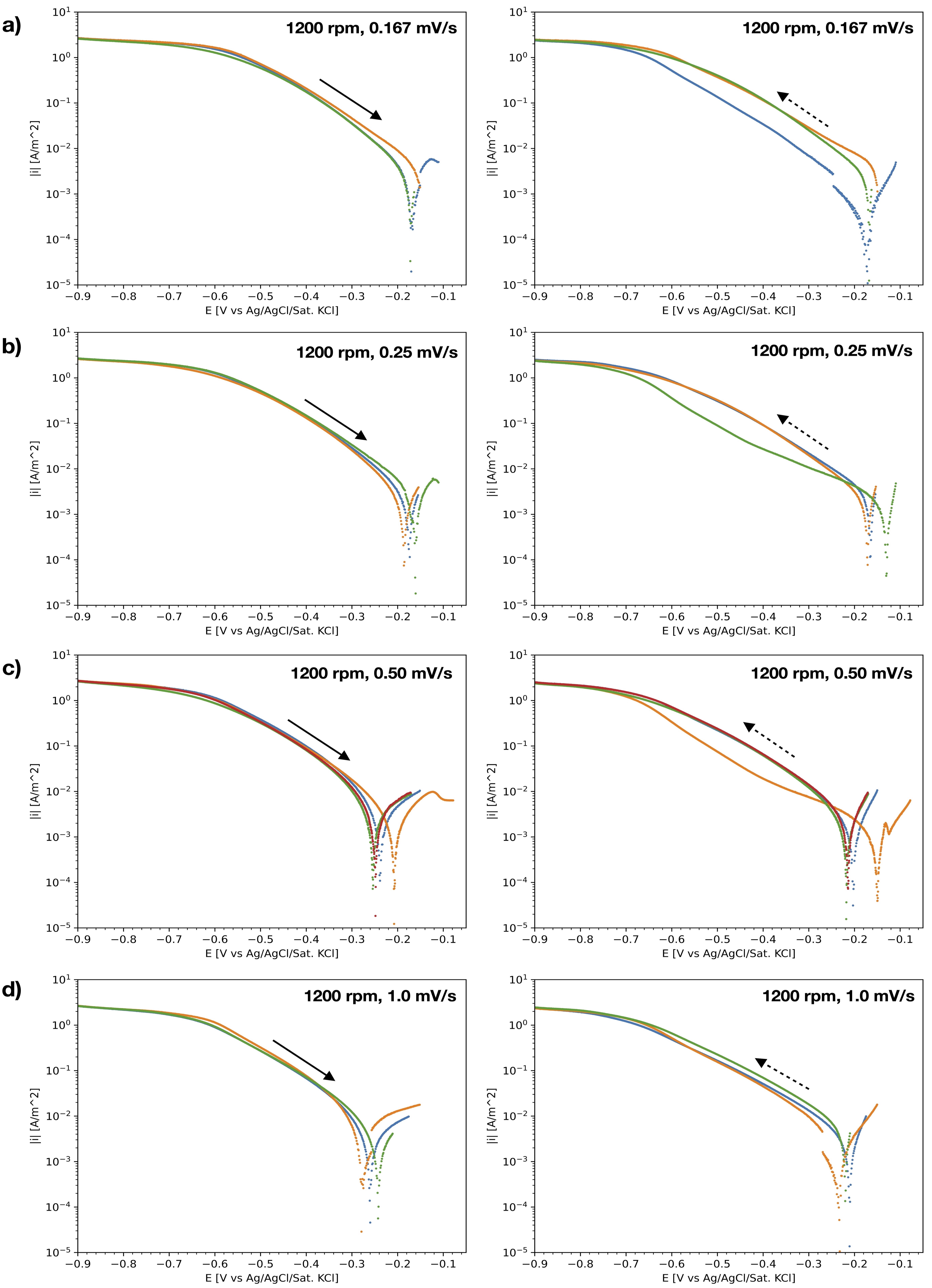


Figure C2. All IR-drop corrected polarization curves, measured with a RDE on stainless steel in an aerated neutral borate buffer solution, for different scan rates: a) 0.167 mV/s, b) 0.25 mV/s, c) 0.5 mV/s, d) 1.0 mV/s (ORR-sr, table 2). Left) the initial upwards scan, right) the following downwards scan.


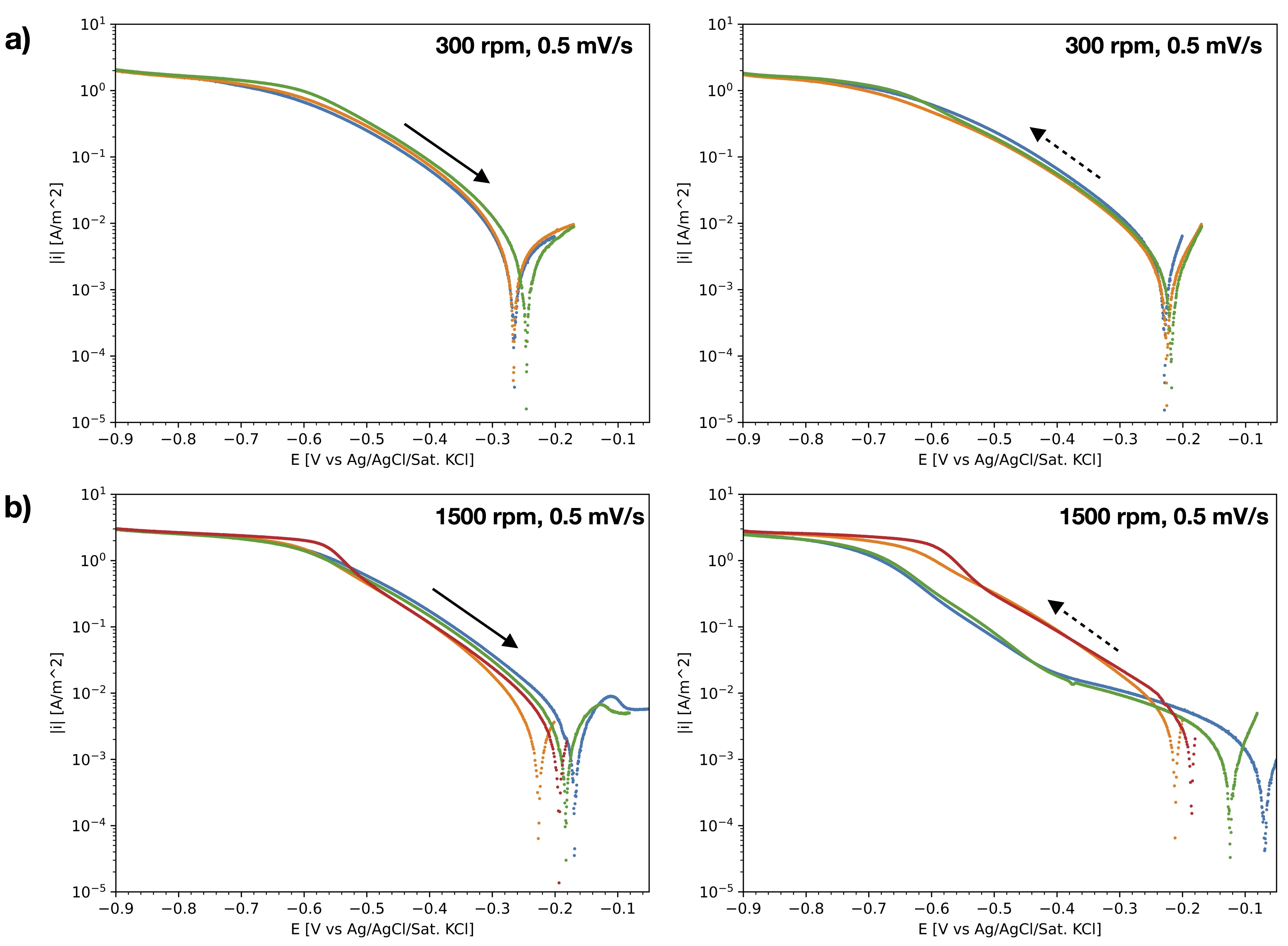


Figure C3. All IR-drop corrected polarization curves, measured with a RDE on stainless steel in an aerated neutral borate buffer solution, for different rotation rates: a) 300 rpm, b) 600 rpm, c) 1500 rpm, d) 1800 rpm (ORR-rr, table 2). Left) the initial upwards scan, right) the following downwards scan.


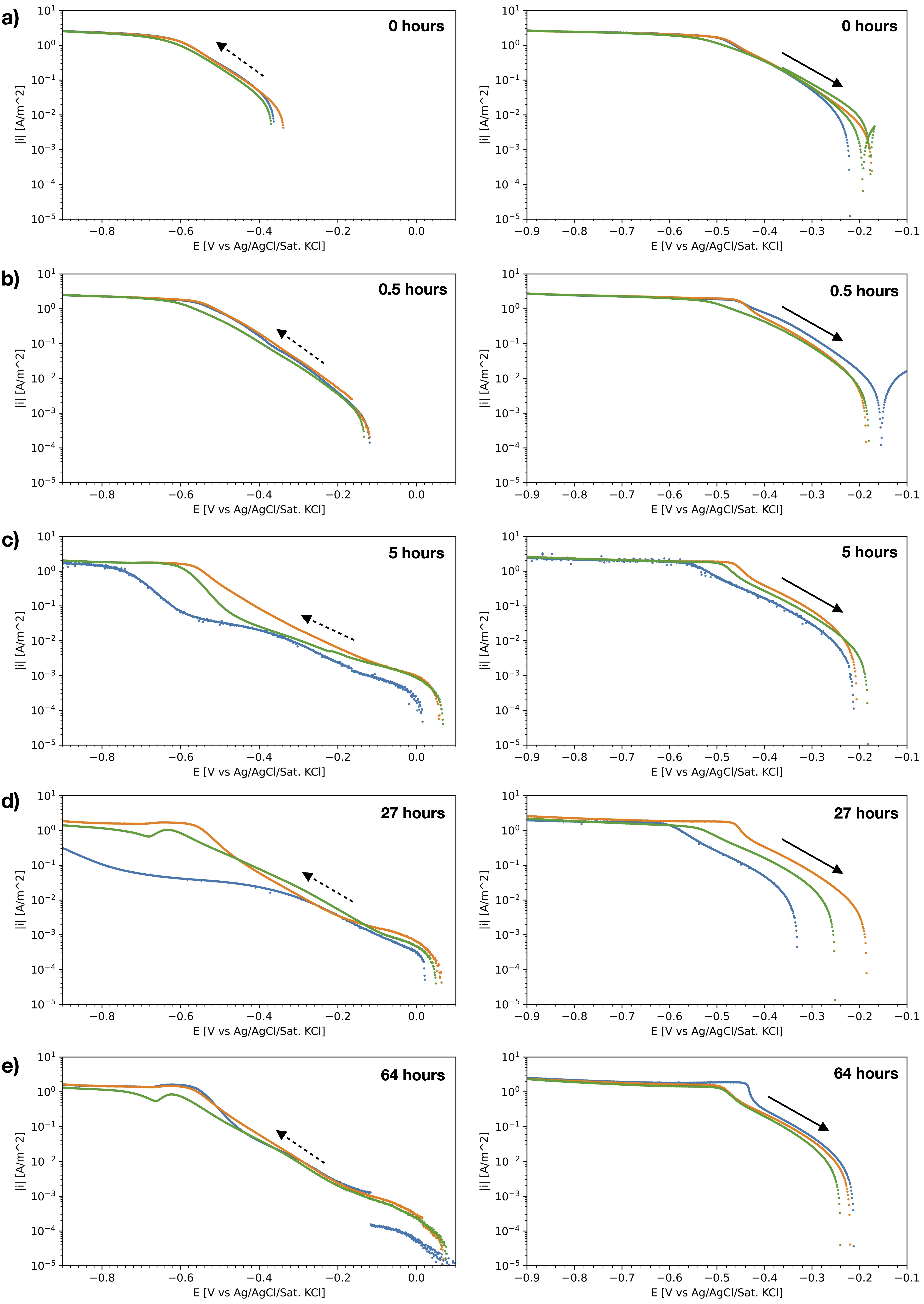


Figure C4. All IR-drop corrected polarization curves, measured with a RDE on stainless steel in an aerated neutral borate buffer solution, for different submerge times: a) 0 h, b) 0.5 h, c) 5 h, d) 27 h, e) 64 h (ORR-st, table 2). Left) the initial upwards scan, right) the following downwards scan.

## C.3 Fitted kinetic parameters

Table C1. Kinetic parameters of the oxygen reduction reaction, obtained by fitting polarization curves measured in an upwards scan direction (-1.5 V vs Ag/AgCl/Sat.KCl –> OCP) on stainless steel in an aerated neutral Borate Buffer (section C.2).

*Table C2. Kinetic parameters of the oxygen reduction reaction, obtained by fitting polarization curves measured in a downwards scan direction(OCP –> -1.5 V vs Ag/AgCl/Sat.KCl) on stainless steel in an aerated neutral Borate Buffer (section C.2).*

Table C3. *Kinetic parameters of the oxygen reduction reaction, obtained by fitting polarization curves measured in an upwards scan direction (-1.5 V vs Ag/AgCl/Sat.KCl –> OCP) on stainless steel in an aerated neutral Borate Buffer, for different submerge times (Figure C4).*

*Table C4. Kinetic parameters of the oxygen reduction reaction, obtained by fitting polarization curves measured in a downwards scan direction(OCP –> -1.5 V vs Ag/AgCl/Sat.KCl) on stainless steel in an aerated neutral Borate Buffer, for different submerge times (Figure C4).*
